# Different life stage, different risks: Thermal performance across the life cycle of *Salmo trutta* and *Salmo salar* in the face of climate change

**DOI:** 10.1002/ece3.7731

**Published:** 2021-06-08

**Authors:** Oskar Kärcher, Martina Flörke, Danijela Markovic

**Affiliations:** ^1^ Faculty of Business Management and Social Sciences Osnabrück University of Applied Sciences Osnabrück Germany; ^2^ Center for Environmental Systems Research University of Kassel Kassel Germany; ^3^ Institute of Hydrological Engineering and Water Management Ruhr‐Universität Bochum Bochum Germany

**Keywords:** climate change, global, life stages, species distribution modeling, species traits, thermal performance curves

## Abstract

Extending assessments of climate change‐induced range shifts via correlative species distribution models by including species traits is crucial for conservation planning. However, comprehensive assessments of future distribution scenarios incorporating responses of biotic factors are poorly investigated. Therefore, the aim of our study was to extend the understanding about the combined usage of species traits data and species distribution models for different life stages and distribution scenarios. We combine global model predictions for the 2050s and thermal performances of *Salmo trutta* and *Salmo salar* under consideration of different life stages (adults, juveniles, eggs), timeframes (monthly, seasonally, yearly), and dispersal scenarios (no dispersal, free dispersal, restricted dispersal). We demonstrate that thermal performances of different life stages will either increase or decrease for certain time periods. Model predictions and thermal performances imply range declines and poleward shifts. Dispersal to suitable habitats will be an important factor mitigating warming effects; however, dams may block paths to areas linked to high performances. Our results emphasize enhanced inclusion of critical periods for species and proper dispersal solutions in conservation planning.

## Graphical abstract

For *Salmo trutta* and *Salmo salar*, we combine species distribution models with thermal performance curves for different life stages, time frames, and dispersal scenarios to assess future impacts of climate change. We show varying thermal performances for different life stages and time periods, range declines, and adverse impacts of dispersal barriers. Our results emphasize enhanced inclusion of critical time periods and consideration of dispersal possibilities.

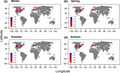

## 1 INTRODUCTION

Temperature is a major factor influencing freshwater fish species distributions, abundances, and physiological rates (Krenek et al., 2011; Schulte et al., 2011). Specifically, water temperature influences the rate of biochemical reactions in aquatic ectothermic species (Angilletta, 2009; Angilletta et al., 2002; Childress & Letcher, 2017; Hochochka & Somero, 2002; Rome et al., 1992) and consequently traits such as growth, development, behavior, metabolic processes, and timing and duration of life‐history events (Huey & Stevenson, 1979; Jonsson & Jonsson, 2009; Jonsson & d’Abée‐Lund, 1993; Scranton & Amarasekare, 2017; Wootton, 1998). With accumulating evidence for increases in the mean temperature and disrupted thermal regimes (Field et al., 2014; Jatteau et al., 2017; Scranton & Amarasekare, 2017; Souchon & Tissot, 2012), the inclusion of functional traits into the analysis of climate change impacts becomes more necessary.

The incorporation of functional traits enables a more reliable assessment of reactions to changing environmental conditions, range shifts, upcoming risks, and new conservation opportunities (Floury et al., 2017; MacLean & Beissinger, 2017; Vasconcelos et al., 2017). Consequently, species traits can be fundamental components for investigating spatially explicit impacts of climate change (MacLean & Beissinger, 2017). Responses of these functional traits along thermal gradients can be parameterized with thermal performance curves (TPCs) (Childress & Letcher, 2017; Jonsson & Jonsson, 2009). Angilletta (2009) defines performance as any measure of an organism's capacity to function. Measures of performance can include, for example, growth, locomotion, or survivorship, which are usually expressed as a rate or probability (Angilletta, 2006, 2009; Schulte et al., 2011). Thermal performance curves (TPCs) are generally used for predicting performance for different thermal environments and inferring the direct effect of temperature on the species’ fitness (Childress & Letcher, 2017; Deutsch et al., 2008; Frazier et al., 2006; Huey & Stevenson, 1979). Thus, the incorporation of functional traits through TPCs and the corresponding thermal tolerance may be promising for exploring range dynamics and species‐specific variation in range shifts under climate change (MacLean & Beissinger, 2017).

Thermal tolerance depends on the physiological sensitivity of the fish to temperature changes and also on the current life stage, with the youngest life phase, that is, the egg stage, being the most susceptible to high and low temperatures and temperature fluctuations (Brett, 1952; Dahlke et al., 2020; Elliott, 1994; Elliott & Elliott, 2010; Jatteau et al., 2017; Jonsson & Jonsson, 2009). Embryonic development is influenced by the surrounding temperature conditions, which additionally affects later species traits and life‐history events, such as smolt size (Jonsson & Jonsson, 2009). Thus, changes in climatic conditions in one life stage can have substantial consequences for later life stages (Fleming et al., 1997; Jonsson & Jonsson, 1993). For example, in early life stages warm temperatures might be favorable for rapid growth whereas in later life stages they might limit growth (Angilletta, 2009). Including different life stages of species with complex life cycles becomes essential in regard to assessing the comprehensive effects of climate change on species.

Assessments of future impacts of climatic change mostly rely on statistical species distribution models (SDMs). There are only a few studies that have combined species functional traits with model predictions for fish species (Wittmann et al., 2016). Wittmann et al. (2016) have found correlations between the probability predictions of the models for habitat suitability and growth rates for the Grass Carp (*Ctenopharyngodon idella*), indicating that SDMs may be able to provide scenarios which incorporate more than just the climatic envelope of the considered species in an indirect manner. Similar results were also observable in studies of other taxonomic groups. For example, Nagaraju et al. (2013) have shown significant positive correlations between the predicted habitat quality and plant functional traits for the endemic tree *Myristica malabarica* Lam. occurring in the Western Ghats in India. To further support these results, more studies that explicitly test SDMs with respect to species traits are necessary.

This study focuses on the salmonid fish species *Salmo trutta* and *Salmo salar*. Anadromous salmonid species have complex life cycles comprising life stages in freshwater, for example, the egg and juvenile stage, and also in saltwater, following the transformation of the species through a smolting process. After living in coastal areas, adults return to their freshwater habitat for spawning (Elliott, 1994). Therefore, effects on one life stage throughout the whole life cycle can substantially affect species traits of the subsequent life stages (Jonsson & Jonsson, 2009), which underlines the necessity for analyses of climate change impacts across life stages of *Salmo trutta* and *Salmo salar*. Also, life stage‐specific analysis enables the detection of shifts of certain life‐history events, such as spawning (Carlson & Seamons, 2008). Although current IUCN Red List conservation status for both species is categorized as “Least Concern” (see https://www.iucnredlist.org/), extinction of southern populations accompanied by a northward movement of the thermal niche in the northern hemisphere is expected (Jonsson & Jonsson, 2009). To facilitate species movement according to the expected geographical shift, accessibility to suitable areas in the future must be guaranteed (Ovidio & Philippart, 2002). Here, we combine species distribution modeling and species’ functional traits to assess future climatic impacts. *Salmo trutta* and *Salmo salar* are analyzed in regard to their current and future thermal performance, here defined as survivorship (Angilletta, 2009), based on the species‐specific derived thermal performance curves (TPCs) (Deutsch et al., 2008). Future thermal performance is deduced from SDM predictions under three different scenarios (“no dispersal,” “free dispersal,” “restricted dispersal”), also accounting for habitat fragmentation due to artificial barriers and connectivity. SDMs are calibrated with global distribution and environmental data at the catchment scale. Specifically, we examine the changes in performance based on the current and predicted distributions across latitude, months, seasons, years, and life stages. Performance parameters are evaluated for the life stages adults, juveniles, and eggs for both study species. Finally, we test whether the probability predictions of the calibrated SDMs in this study are correlated with the performance rates of the TPCs (see Wittmann et al., 2016).

## 2 METHODS

### 2.1 Study area

Global land masses were divided into African, Asian, Australian, European, North American, and South American regions (Figure S1). Each region was additionally differentiated into sub‐watershed basins (bas20k) based on the utilization of the integrated water resource model WaterGAP3 (Brauman et al., 2016; Eisner, 2016; Schneider et al., 2017). To reduce uncertainty of environmental data calculations, only catchments with an area of ≥3,000 km² were included. The total global catchment number was 11,695 (Table S1). Analyses were restricted to freshwater habitats, because of the studied catchment scale, prevailing occurrences of the investigated species in fresh waters and greater threats of climate change and anthropogenic disturbances to fresh waters rather than marine realms (Dudgeon et al., 2006; Pachauri & Mayer, 2015).

### 2.2 Species data

Global species occurrence data for *Salmo trutta* and *Salmo salar* were obtained from the Global Biodiversity Information Facility (https://www.gbif.org/, accessed November 2018), which provides occurrence data via longitudinal and latitudinal specifications. We considered only presences from 1971 onwards with a coordinate uncertainty of ≤5 km and with “human observation” set as basis of record. Freshwater species data were mapped to the bas20k catchments leading to in total 730 and 199 catchment occurrences for *Salmo trutta* and *Salmo salar*, respectively (Table S1). In the case of coarser scales or the creation of range maps, which are expected to be less spatially biased (Fourcade, 2016; Merow et al., 2016), the number of false absences will diminish rapidly due to the diminution in the degree of patchiness, that is, the mapping to coarser modeling scales overcomes potentially neglected occurrence points and aims at reducing the sampling bias. Therefore, by choosing a coarse catchment scale, the effect of such errors is minimized and consequently the likelihood for true absences is increased (see Rocchini et al., 2011). Furthermore, in comparison with point‐to‐grid mapping used for mapping terrestrial species’ occurrences, catchment mapping is more appropriate for freshwater species due to the dendritic structure of river networks (see Fagan, 2002). Catchment assessments are used for large‐scale freshwater management strategies (commonly referred to as the Catchment‐Based Approach—CaBA, see DEFRA, 2013), enabling the compatibility between the management and the analysis scales as well as the optimization of ecological restoration efforts (Lévêque et al., 2008; Markovic et al., 2017; Kümmerlen et al., 2019).

### 2.3 Environmental data

Modern‐day (1971–2000, hereafter referred to as baseline) and future (2041–2070, hereafter referred to as 2050s) data on natural river discharge were obtained from the WaterGAP3 model (Brauman et al., 2016; Eisner, 2016; Schneider et al., 2017). WaterGAP3 is a state‐of‐the‐art global water model showing well performance (Beck et al., 2017; Eisner et al., 2017; Schneider et al., 2017). Grid‐based monthly water balance calculations of WaterGAP3 at the 5 by 5 arc‐minute resolution (~9 × 9 km at the Equator) were mapped to the bas20k catchment scale and used for catchment‐specific calculations of various single‐value discharge statistics for the baseline and 2050s (Table S2). Future flow statistics were computed as multimodel ensemble means of five different general circulation models (GCMs), namely GFDL‐ESM2M, HadGEM2‐ES, IPSL‐CM5A‐LR, MIROC‐ESM‐CHEM, and NorESM1‐M, provided by ISI‐MIP (Hempel et al., 2013). Each GCM followed the medium–high emission Representative Concentration Pathway 6.0 (RCP6.0) scenario, which comprises a radiative forcing of 6.0 W/m^2^ in the year 2100 and a global mean temperature increase of 2.2°C until the end of the century compared with 1986–2005 (Riahi et al., 2011). Accordingly, the five integrated GCMs were used to derive water temperature by transforming air temperature to stream water temperature on a monthly basis for the baseline and 2050s via a global relationship model (Punzet et al., 2012) (Table S2).

We used the Global Land Cover Characterization map (GLCC; USGS, 2008) and the CORINE Land Cover map for EU countries (CLC2000; EEA, 2004) for obtaining landscape variables (Table S2). Land cover data were kept constant for the future scenario.

In order to model species distribution opportunities and restrictions, we included the Global Reservoir and Dams database (GRanD) (Lehner et al., 2011).

### 2.4 Species traits data

We collected laboratory experiment data on species thermal traits for *Salmo trutta* and *Salmo salar* at different life stages from various studies (Tables S3 and S4). Data could be collected for the traits “critical minimum temperature for survival” (CT_min_), “optimum temperature” (*T*_opt_), “critical maximum temperature for survival” (CT_max_) and for the life stages adults, juveniles, and eggs. The thermal optimum (*T*_opt_) was defined as the upper limit of the optimum temperature range following Comte et al. (2014). The maximum/ minimum temperature for survival was determined using different experimental approaches, such as the incipient lethal temperature (ILT) method or the critical thermal methodology (CTM). The incipient upper/lower lethal temperature (IULT/ ILLT) is defined as the temperature that is lethal to 50% of a fish sample estimated over various acclimation temperatures and exposure time intervals whereas for CTM the critical maximum/ minimum temperature is determined by exposing species to a constant linear increase or decrease in temperature until the fish loses its locomotion control (Beitinger et al., 2000). Moreover, a recent study by O'Donnell et al. (2020) has proven that the critical maximum temperature determined with CTM appears to be a robust, repeatable estimate of thermal tolerance in cold‐water adapted fish. The maximum and minimum of the experimentally observed CT_min_ and CT_max_, respectively, were set as final CT_min_ and CT_max_. Although experimental values could be collected from various studies, species trait data remain limited. Moreover, TPCs may not be fixed within species or individuals but can change as a result of adaptation and epigenetic processes in response to temperature signals at different time scales. Hence, the conclusions drawn in this study should be seen in the context of the collected trait data.

### 2.5 Thermal performance curves

Species thermal traits data were used to parametrize thermal performance curves (TPCs), which describe the relationship between temperature and a species’ ability to function (Angert et al., 2011; Huey & Stevenson, 1979). Typically, TPCs are bounded at the extreme temperatures (CT_min_, CT_max_), possess a single intermediate mode, and appear skewed with a slow performance rise up to the maximum level at *T*_opt_ and a rapid drop afterward (Angilletta, 2006; Dell et al., 2011; Huey & Kingsolver, 1989; Sinclair et al., 2016). The skewness of the TPCs arises from slower chemical reactions at low temperatures and constraints of the cellular function capacity due to protein degradation and oxygen limitation at high temperatures (Childress & Letcher, 2017; Dell et al., 2011). Here, performance is defined as survivorship given as a rate along the thermal gradient (Angilletta, 2009). The model of Deutsch et al. (2008) was used to obtain the performance rates by incorporating the observed data on CT_min_, T_opt_, and CT_max_ for the life stages adults, juveniles, and eggs, thus, accounting for varying TPCs and stage‐specific vulnerability (Sinclair et al., 2016).

### 2.6 Species distribution modeling

The predictor variable selection was based on a combination of three main criteria: (1) the univariate area under the receiver operating characteristic curve (AUC ≥0.65), (2) avoidance of multicollinearity (pairwise correlations of <0.7), and (3) variable selections in previous studies. Univariate prediction strength was determined by using generalized additive models (GAMs) of the R (R development Core Team, 2018) package “mgcv” (Wood, 2011) as modeling approach. Often‐used variables incorporated in scientific literature were only included if at least the second criterion was fulfilled.

Fish distributions were modeled using Artificial Neural Networks (ANN), Random Forest (RF), Gradient Boosting Machines (GBM), Multivariate Adaptive Regression Splines (MARS), Generalized Additive Models (GAM), Maximum Entropy Method (MAXENT), and Elastic Net (ELNET). ANNs are complex, nonlinear model systems resembling the biological neural system, that is, ANNs include neurons with a specified number of layers that are linked by different types of so‐called activation functions (Bishop, 1995; Duda et al., 2001; Hastie et al., 2001; Jain et al., 1996; Lee et al., 2016; Li & Wang, 2013). Commonly, a three‐layer feedforward model is used, which consists of the input layer, the hidden layer, and the output layer (Bishop, 1995) and which can approximate any smooth, finite nonlinear function with high accuracy (He et al., 2011; Thuiller et al., 2009). We used the R package “h2o” (The H2O.ai team, 2018) for training ANNs as it provides many opportunities to adapt the model to the specific problem. RF is a combination of a certain number of decision trees where each tree is created by considering a random sample of the training data set and features (Breiman, 2001). The number of votes of each tree of the forest determines the final prediction. High performances in species distribution modeling can be achieved by using this learning algorithm (e.g., Grenouillet et al., 2011). For building RF, we used the R package “h2o” (The H2O.ai team, 2018). GBMs consist of a group of decision trees, which are build and combined by the gradient boosting algorithm (Elith et al., 2008; Hastie et al., 2001). Here, the R package “h2o” (The H2O.ai team, 2018) was used for analyses. MARS is a flexible regression method based on piecewise splines that are smoothly connected and thus able to model linear and nonlinear relationships (Friedman, 1991; Zhang & Goh, 2016). The R package “earth” was used for MARS modeling (Milborrow, 2018). GAM is a nonparametric method that is able to account for nonlinear relationships between the explanatory and dependent variables by using smoothing functions (Hastie & Tibshirani, 1986). For GAM, we implemented the function of the R package “mgcv” (Wood, 2011). MAXENT as a general‐purpose machine learning method is a principle from statistical mechanics and information theory (Phillips et al., 2006). It uses only presence data to estimate a target probability distribution by finding the probability distribution of maximum entropy under the constraint of the original data properties (Phillips & Dudik, 2008). The package “dismo” of Hijmans et al. (2017) was employed for the utilization of MAXENT. ELNET, which consists of a generalized linear model with a Lasso and Ridge regularization (L1 and L2 regularization) (Friedman et al., 2010), was used from the R package “h2o” (The H2O.ai team, 2018).

In order to tailor the models to our specified modeling problem, we conducted a hyperparameter tuning for the statistical methods ANN, RF, GBM, MARS, and ELNET. The “h2o” package offers many tuning options for ANN with the possibility of manual tuning of the learning rates and momentum as well as the possibility of using the ADADELTA method (adaptive learning rate method) of Zeiler (2012). Manual and ADADELTA parameter tuning followed the instructions of the “h2o” manuals (https://www.h2o.ai/resources/, accessed October 2018). Parameter tunings for RF, GBM, and ELNET from the R package “h2o” (The H2O.ai team, 2018) were also carried out according to “h2o” guidelines. For MARS, only the tuning parameter for the maximum number of terms was tuned (Milborrow, 2018; Zhang & Goh, 2016). All tuning parameters are summarized in Table S5. The hyperparameter optimization strategy for manual and ADADELTA ANN, RF, GBM, and ELNET was random grid search (*n* = 300) since random parameter combination search was shown to find good or even better models compared with pure grid search within a small fraction of the computation time (Bergstra & Bengio, 2012). However, all parameter possibilities were tested for MARS due to significantly less computational cost. For each model that required parameter tuning, we estimated the best parameter combination using the threshold independent performance measure “AUC” resulting from fivefold cross‐validation of 80% of the data (Bergstra & Bengio, 2012; El‐Gabbas & Dormann, 2017). The remaining 20% were withheld to simulate performance testing on an unseen and independent data set (Bergstra & Bengio, 2012). For the two ANNs, only the model with better performance on the test data set was used in further analyses.

Accuracy of various predictive performance measures after final parameter determination was tested by randomly splitting the data into 80% calibration and 20% validation data 100 times. A threshold for the probability predictions, that is, for separating presences and absences of a species, was determined by minimizing the absolute difference between specificity (the rate of correctly predicted absences) and sensitivity (the rate of correctly predicted presences) (Fielding & Bell, 1997). Minimizing the difference between the sensitivity and specificity generally leads to accurate predictions (Jimenez‐Valverde & Lobo, 2007). Therefore, we also considered the threshold‐dependent performance measure “true skill statistic” (TSS = sensitivity + specificity – 1). Validation performance results for AUC, sensitivity, specificity, and TSS were computed each time, whereas the average validation performance was used for the assessment of the predictive performance (Dormann et al., 2008).

For species distribution predictions, we applied the consensus method by averaging the resulting probabilities of occurrence in order to reduce uncertainty of using a single modeling approach (Marmion et al., 2009). To ensure reliability and robustness of our statistical approaches, only those models with a mean validation AUC >0.85 were included. Following this validation approach, we also conducted a validation of the performance measure accuracy for the ensemble model.

We studied three different scenarios in the future spatial distribution patterns of the considered species in terms of distribution possibilities. The first possibility considers no change in future distribution ranges (“no dispersal”), that is, the 2050s distribution range corresponds to the range of the baseline in order to identify affected areas in the future of current distributions. The second possibility comprises a free distribution of the species on condition that the predicted presence in a catchment is connected to a catchment with a presence in the baseline pattern (“free dispersal”). The third possibility is defined by a restricted distribution of the species, which considers dams as dispersal barriers (“restricted dispersal”), in order to outline the effects of habitat fragmentation on future species distributions and performance.

### 2.7 Assessment of species’ thermal performance

Thermal performance analyses were based on the baseline and future (“no dispersal,” “free dispersal,” “restricted dispersal”) distribution data, water temperature data, and the parametrized TPCs for the different life stages (adult, juveniles, and eggs) (see Figure S2). Predictions by SDMs were constructed per species prior to thermal performance analyses, which were carried out post hoc for each life stage and different timeframes; such that for every predicted occurrence, a thermal performance measure was calculated. Species performance was studied at a monthly, seasonally, and yearly timeframe. Monthly and seasonal analyses were performed for capturing potential phenology shifts either due to enhanced or reduced fitness (Deutsch et al., 2008). Seasons were defined as winter (December–February), spring (March–May), summer (June–August), and autumn (September–November) according to the northern hemisphere. The spawning season for eggs in the northern hemisphere was defined as October–February (Campbell, 1977; Elliott & Elliott, 2010; Jonsson & Jonsson, 2009, 2011, 2014; Östergren & Rivinoja, 2008) and for the southern hemisphere as April–August, being six months out of phase with northern conspecifics (Pankhurst & King, 2010). A broad spawning season was chosen to cover phenotypical divergence across populations (Angert et al., 2011; Hereford, 2009). Seasonal and yearly estimates were based on previously calculated monthly performances. Additionally, latitudinal distributions of the performance rates were investigated in order to understand how the thermal performance might change (Sinclair et al., 2016). The main workflow is summarized in Figure S3.

Relationships between modeled habitat suitability given as a probability for the baseline and the species’ functional trait expressed as the thermal performance were quantified via a correlation analysis to test the implicit assumption of SDMs that highly suitable sites with high probabilities of occurrence imply higher performance and fitness than poorly suitable sites with lower probabilities of occurrence (Guisan & Thuiller, 2005; Wittmann et al., 2016). Thus, the concept of the environmental niche modeling, which commonly uses abiotic conditions for explaining species distributions, is examined by relating to a biotic factor.

## 3 RESULTS

### 3.1 Predictor variable selection

Through synthesis of three variable selection criteria (univariate analysis, correlation analysis, and scientific literature), we selected 8 from 29 variables representing climatic, topographic, and anthropogenic influences from the baseline data set for each species (Table 1, Tables S6–S7, Figure S4). The variable selection accounted for seasonal discharge and water temperature influences in regard to spawning seasons. Discharge variables were all highly correlated with each other, thus limiting the number of discharge variables in the model (Figure S4). For example, we explained *Salmo trutta* distributions by selecting the Mean autumn water temperature because of the combination of univariate explanatory strength and approaches in scientific literature (Tables S2 and S6). Due to high pairwise correlations of the mean autumn water temperature with other water temperature variables, only the annual water temperature range and mean diurnal range were additionally included. Mean winter discharge was analogously selected because of the combination of univariate explanatory strength and its influence on specific life stages. Factors of anthropogenic and topographic influences were taken into account through built‐up area, forest, cropland, and altitude, whereas especially cropland and altitude were included due to scientific literature.

**TABLE 1:** Variable selection for modeling species distributions of *Salmo trutta* and *Salmo salar*

Category	Variable	Description	Species	
			*Salmo trutta*	*Salmo salar*
Climatic	Mean winter discharge	Mean discharge for the months December–February	Yes	Yes
	Water temperature seasonality	Average of the annual standard deviation of water temperatures	No	Yes
	Mean autumn water temperature	Mean water temperature for the months September–November	Yes	Yes
	Mean diurnal range	Mean of monthly (maximum–minimum water temperature)	Yes	No
	Annual water temperature range	Maximum water temperature–minimum water temperature	Yes	No
	Isothermality	Mean diurnal range / Annual water temperature range	No	Yes
Topographic	Altitude	Mean catchment elevation	Yes	Yes
Land cover	Cropland	Percentage of catchment area covered by cropland	Yes	Yes
	Built‐up area	Fraction of sealed areas within the catchment	Yes	Yes
	Forest	Percentage of catchment area covered by forest	Yes	Yes

### 3.2 Model performance

Cross‐validated AUC values and AUC scores for the test data set during parameter tuning showed high performances (AUC >0.90) (Tables S8 and S9) with only ELNET having lower performance scores (AUC <0.87) for both species. Final parameter tuning results are listed in Appendix S1 (Tables S8 and S9). Differences between the AUC test scores, although small, of the manually and ADADELTA tuned ANNs, led to the further inclusion of the manually tuned ANN model for *Salmo trutta* (Table S8) and the ADADELTA ANN for *Salmo salar* (Table S9). The performance validation showed high mean scores (e.g., mean AUC ≥0.95) after parameter tuning for all included statistical models except for ELNET (mean AUC ≤0.85) (see Table 2). Additionally, threshold‐dependent performance measures, that is, sensitivity, specificity, and TSS, attained high values for nearly all statistical approaches. Medium performance values (e.g., 0.7 ≤ AUC ≤ 0.9) were only found for ELNET (Table 2). Thus, ELNET was excluded in the ensemble modeling for both species. The validation performance values of the consensus models were in the range of the high values of each included statistical model (Table 2).

**TABLE 2:** Validation performance results of all considered statistical methods (Artificial Neural Networks (ANN), Random Forest (RF), Gradient Boosting Machines (GBM), Multivariate Adaptive Regression Splines (MARS), Generalized Additive Models (GAM), Maximum Entropy Method (MAXENT), Elastic Net (ELNET), and consensus method (CONS))

Species	Performance measure		Method							
			ANN	RF	GBM	MARS	GAM	MAXENT	ELNET	CONS
*Salmo trutta*	AUC	Min	0.97	0.97	0.97	0.93	0.94	0.95	0.80	0.97
		Mean	0.98	0.98	0.98	0.95	0.96	0.96	0.82	0.98
		Max	0.99	0.99	0.99	0.96	0.97	0.97	0.84	0.98
	Sensitivity	Min	0.91	0.92	0.92	0.85	0.87	0.86	0.72	0.90
		Mean	0.93	0.94	0.94	0.88	0.90	0.90	0.74	0.92
		Max	0.95	0.95	0.96	0.91	0.92	0.93	0.76	0.94
	Specificity	Min	0.91	0.92	0.91	0.85	0.87	0.86	0.72	0.90
		Mean	0.93	0.94	0.94	0.88	0.90	0.93	0.74	0.92
		Max	0.95	0.95	0.96	0.92	0.92	0.90	0.76	0.94
	TSS	Min	0.82	0.84	0.83	0.69	0.75	0.73	0.44	0.81
		Mean	0.86	0.87	0.87	0.76	0.79	0.80	0.48	0.84
		Max	0.90	0.91	0.92	0.83	0.84	0.85	0.53	0.88
*Salmo salar*	AUC	Min	0.95	0.97	0.95	0.93	0.94	0.96	0.81	0.96
		Mean	0.97	0.98	0.98	0.96	0.97	0.97	0.85	0.98
		Max	0.99	0.99	0.99	0.98	0.98	0.99	0.89	0.99
	Sensitivity	Min	0.86	0.87	0.86	0.85	0.83	0.86	0.74	0.86
		Mean	0.91	0.93	0.93	0.90	0.91	0.92	0.78	0.92
		Max	0.95	0.97	0.97	0.94	0.95	0.98	0.83	0.97
	Specificity	Min	0.87	0.90	0.88	0.85	0.84	0.86	0.73	0.87
		Mean	0.91	0.93	0.93	0.90	0.91	0.92	0.78	0.92
		Max	0.96	0.97	0.97	0.94	0.95	0.97	0.82	0.96
	TSS	Min	0.73	0.79	0.73	0.70	0.67	0.72	0.47	0.73
		Mean	0.82	0.87	0.86	0.79	0.82	0.83	0.56	0.84
		Max	0.91	0.94	0.94	0.89	0.90	0.95	0.64	0.92

Note

For *Salmo trutta*, the manually tuned ANN was used for further analyses, while for *Salmo salar*, the ADADELTA ANN was used. Due to a mean validation AUC of ≤0.85, ELNET was excluded in the consensus model for both species.

### 3.3 Current and future species’ thermal performance

For the thermal performance assessment of the two salmonids, three future dispersal scenarios were considered. While the location and number of presences for the “no dispersal” scenario correspond to the initial baseline situation (*n* = 730 for *Salmo trutta* and *n* = 199 for *Salmo salar*), the remaining two future dispersal scenarios showed a reduction in the distribution ranges. For *Salmo trutta*, *n* = 724 future presences were predicted with the ensemble modeling approach. In regard to the “free dispersal” scenario, which required a connection of the catchment with a predicted presence to a catchment with a baseline presence, the number of predicted presences decreased to *n* = 582 (~−20%), with 464 out of the 730 currently suitable catchments being suitable in the future. The integration of the “restricted dispersal” scenario, which included dams as dispersal barriers, indicated a further decline of the distribution range, with *n* = 475 (~−35%) remaining predicted presences for the 2050s (see Figure S5). For *Salmo salar*, the consensus model predicted *n* = 194 presences for the 2050s. Under the “free dispersal” scenario, the predicted number of presences decreased to *n* = 119 (~−40%) with 89 out of the 199 currently suitable catchments being still suitable in the future. Similarly, the “restricted dispersal” scenario led to a further decline in the number of presences to *n* = 102 (~−49%) (see Figure S6).

**FIGURE 1:**
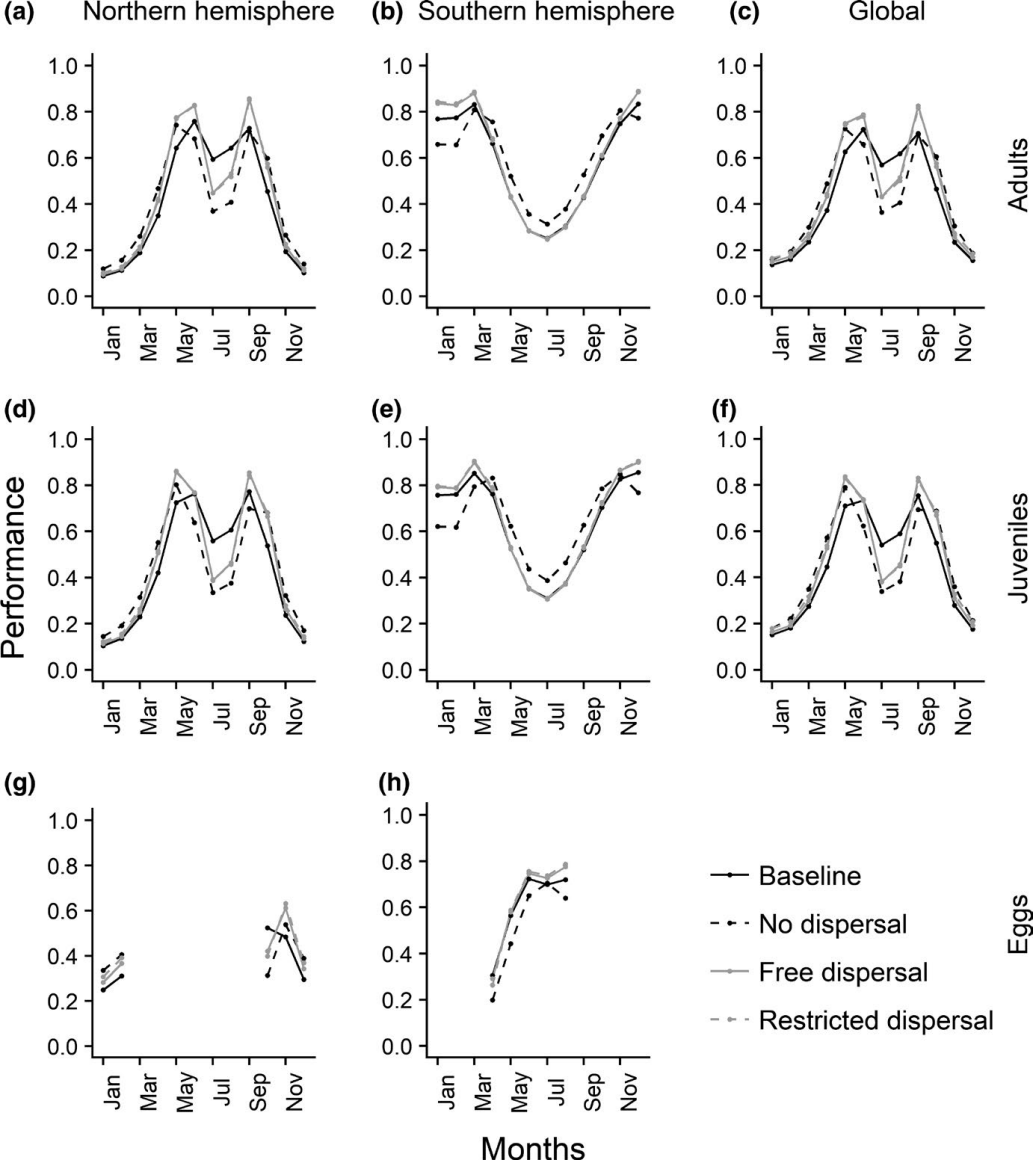
Baseline and 2050s monthly thermal performance given as a rate of survivorship for the life stages adults, juveniles, and eggs of *Salmo trutta* under consideration of different dispersal scenarios

Monthly thermal performance trends for the three life stages were considered separately for the northern and southern hemisphere to account for the shifted seasons (Figure 1 and Figure S7). Mean performances for the life stages of adults and juveniles of *Salmo trutta*, respectively, showed similar monthly trends (Figure 1). We note that less occurrences were present for the southern hemisphere and that results should be interpreted with caution (see Table S1). For the sake of simplicity, the following results focus on the species in the northern hemisphere. Compared with the baseline performance, a slight increase in performance for all dispersal scenarios could be observed from January to June and from September to November, whereas for the remaining months in summer (July, August) all scenarios predicted a drop below the current performance (Figure 1a,d). Future mean performances of the scenarios “free dispersal” and “restricted dispersal” were similar, although the latter scenario led to fewer predicted occurrences. Additionally, both indicated higher performance values from May to September compared with those found under the “no dispersal” scenario. Future monthly performances of eggs during the spawning season showed in general higher performances with a shift of the peak performance from October to November (Figure 1g). For all scenarios of the 2050s, a drop below the current performance was observable in October. Monthly performances for *Salmo salar* were only identified for populations in the northern hemisphere (Figure S7), because low occurrence numbers were present for southern conspecifics (see Table S1). In general, all future scenarios predicted a similar increase in monthly performances for *Salmo salar* adults and juveniles (Figure S7). For eggs, the same monthly performance pattern as found for northern *Salmo trutta* eggs emerged.

**FIGURE 2:**
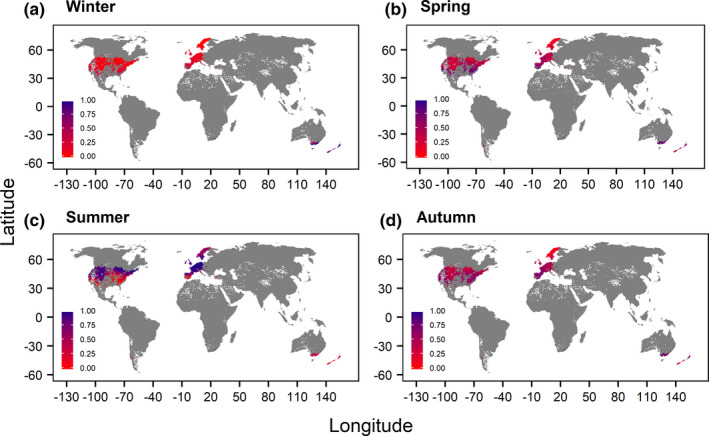
Global map of the seasonal performances of adult *Salmo trutta* for the “baseline” scenario. Note that seasons were defined according to the northern hemisphere

**FIGURE 3:**
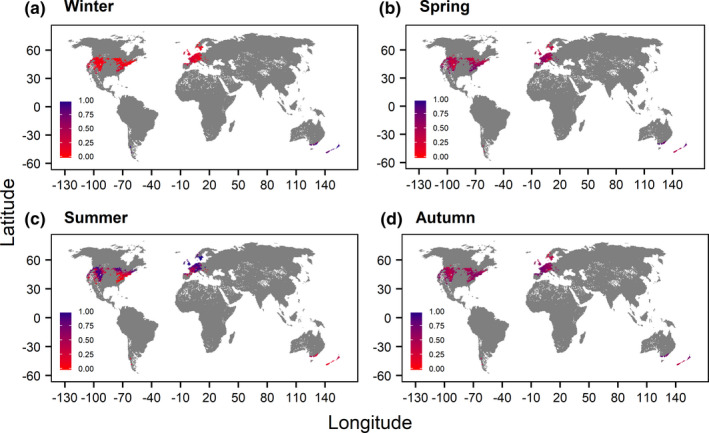
Global map of the seasonal performances of adult *Salmo trutta* for the “restricted dispersal” scenario. Note that seasons were defined according to the northern hemisphere

Seasonal performance patterns of *Salmo trutta* underlined the decrease in the summer performance of adults and juveniles for all scenarios in the southern ranges of the northern hemisphere (Figures 2 and 3, Figures S8–S13 and Table 3). Summer mean performances were higher for the “free” and “restricted dispersal” scenario (adults: 0.60, juveniles: 0.54) compared with the “no dispersal” scenario (adults: 0.49, juveniles: 0.45) (Table 3). Mean performances of northern populations across the winter, spring, and autumn season showed a slight shift from lower to higher performances. The scenario “restricted dispersal” revealed limited distribution possibilities in north‐eastern USA and eastern Europe (Figure 3 and Figure S9). Thus, in northern USA areas where high performance values could be attained in summers of the 2050s could not be reached due to the existence of artificial barriers. Seasonal mean performances of *Salmo trutta* eggs indicated in general increasing performance values for all scenarios (Table 3). Seasonal mean performances of northern *Salmo salar* populations increased similarly for every future scenario and life stage (Table 3, Figures S14–S21). Nonexisting differences between the seasonal mean performances inferred from the “free” and “restricted dispersal” scenario indicated the necessity of geographical inspections. However, for adults and juveniles only the disappearance of catchments connected to high performances could be observed under “restricted dispersal” especially for the summer season in northern Europe and parts of the USA (Figures S16, S17, S20 and S21), which could be ascribed to the small difference (*n* = 17) between the numbers of predicted occurrences of the two scenarios.

**TABLE 3:** Comparison of the mean baseline and future thermal performance as rate for different scenarios and timeframes

Species	Life stage	Timeframe	Scenario							
			Baseline		No dispersal		Free dispersal		Restricted dispersal	
			NH	SH	NH	SH	NH	SH	NH	SH
*Salmo trutta*	Adults	Winter (summer)	0.10	0.79	0.14	0.70	0.11	0.85	0.12	0.86
		Spring (autumn)	0.39	0.64	0.49	0.70	0.46	0.66	0.47	0.67
		Summer (winter)	0.66	0.28	0.49	0.35	0.60	0.28	0.60	0.28
		Autumn (spring)	0.46	0.59	0.53	0.68	0.54	0.60	0.55	0.61
		Annual	0.40	0.58	0.41	0.60	0.43	0.60	0.43	0.60
	Juveniles	Winter (summer)	0.12	0.79	0.17	0.67	0.13	0.83	0.14	0.83
		Spring (autumn)	0.46	0.71	0.56	0.75	0.54	0.73	0.54	0.74
		Summer (winter)	0.64	0.35	0.45	0.43	0.54	0.34	0.54	0.35
		Autumn (spring)	0.51	0.68	0.57	0.75	0.59	0.70	0.60	0.71
		Annual	0.43	0.63	0.44	0.65	0.45	0.65	0.46	0.66
	Eggs	Spawning	0.37	0.60	0.40	0.53	0.41	0.62	0.42	0.63
*Salmo salar*	Adults	Winter (summer)	0.08	–	0.11	–	0.11	–	0.11	–
		Spring (autumn)	0.27	–	0.36	–	0.33	–	0.33	–
		Summer (winter)	0.80	–	0.83	–	0.89	–	0.89	–
		Autumn (spring)	0.35	–	0.46	–	0.42	–	0.43	–
		Annual	0.38	–	0.44	–	0.44	–	0.44	–
	Juveniles	Winter (summer)	0.07	–	0.09	–	0.09	–	0.09	–
		Spring (autumn)	0.21	–	0.29	–	0.25	–	0.26	–
		Summer (winter)	0.70	–	0.83	–	0.79	–	0.79	–
		Autumn (spring)	0.28	–	0.38	–	0.34	–	0.34	–
		Annual	0.32	–	0.40	–	0.37	–	0.37	–
	Eggs	Spawning	0.32	–	0.37	–	0.38	–	0.40	–

Note

Performances for populations in the northern (NH) and southern hemisphere (SH) were computed separately. Performance was identified for the life stages adults, juveniles, and eggs, whereas performance of eggs was only considered within the spawning season of the salmonids *Salmo trutta* and *Salmo salar*. Note, that values for the southern hemisphere of *Salmo salar* were excluded because of few observations. Seasons for the southern hemisphere are given within brackets.

**FIGURE 4:**
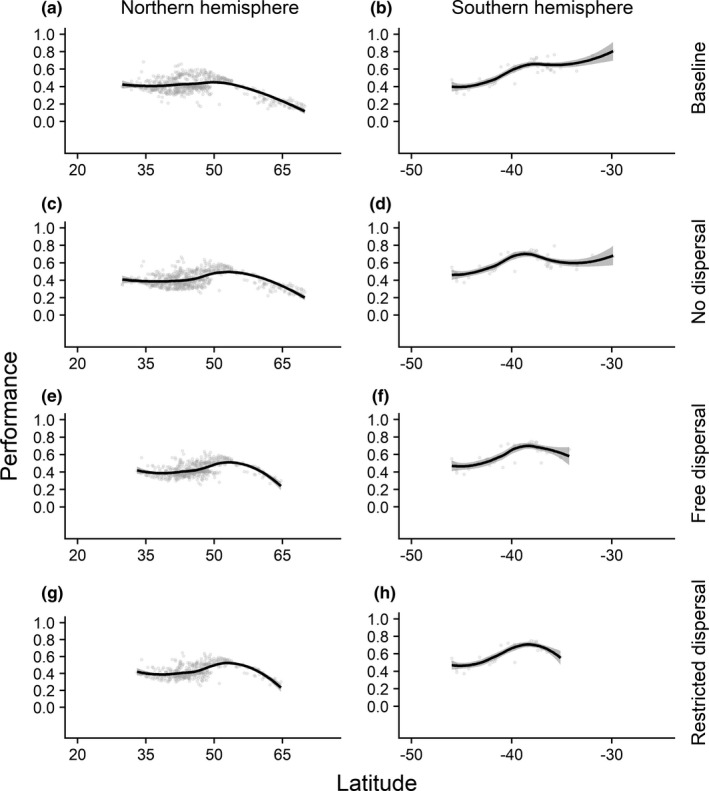
Latitudinal trends of annual mean performance for adult *Salmo trutta* under consideration of different dispersal scenarios. Annual mean performance is based on monthly performance values

Latitudinal trends of the annual mean performance revealed poleward trends across all scenarios and life stages of *Salmo trutta* (Figure 4 and Figures S22–S23). Northward trends were observable due to higher performances around 45°–55°N (northern USA and central Europe) in the future and range shifts identified by the “free” and “restricted dispersal” scenario (e.g., Figure 4e,g). However, the increases in performance up to 55°N were followed by declines in the annual mean performance for more northern or polar regions. The spawning season performance of *Salmo trutta* eggs in these regions dropped even stronger than the performance of adults and juveniles (Figure S23). In general, these observations could be made for the southern hemisphere vice versa. The observed northward trends of the performances and distributions found for *Salmo trutta* in the northern hemisphere could be transferred to all life stages of *Salmo salar* (Figures S24–S26). Future annual and spawning season mean performances increased around 45°–55°N and were accompanied by in part steep negative slopes when moving toward higher latitudes. Trends for the southern hemisphere could not be studied because of low occurrence numbers.

### 3.4 Species distribution models and thermal performance

**FIGURE 5:**
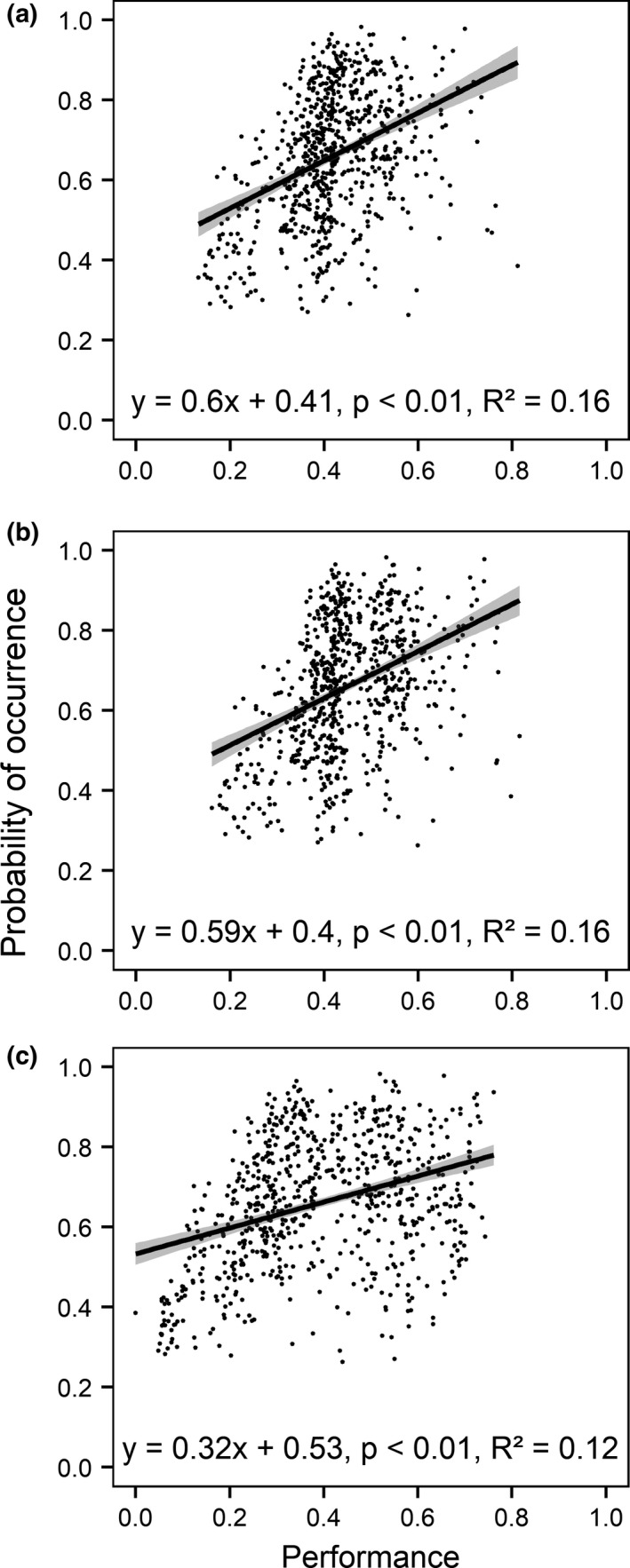
Relationship between the modeled habitat suitability and performance for the life stages (a) adults, (b) juveniles, and (c) eggs of *Salmo trutta*. For adults and juveniles, the annual mean performance and, for eggs, the performance during the spawning season were used for the assessment

Relationships between the modeled probabilities of occurrence and thermal performances of the two salmonids and the respective life stages were investigated in order to test the ability of SDMs to incorporate biotic characteristics through abiotic predictors. The investigation revealed significant positive relationships for all life stages of *Salmo trutta* (*p* < .01; *r* = .40 for adults and juveniles; *r* = .35 for eggs) (Figure 5). However, no significant relationships were found for *Salmo salar* adults and juveniles (*p* > .40). Only between the performance of *Salmo salar* eggs in the spawning season and the probability for a species’ presence a significant positive relationship was identified (*p* < .01; *r* = .28).

## 4 DISCUSSION

Comprehensive assessments of future climate change impacts on species require not only the investigation of abiotic relationships of the species with the environment by using species distribution models (SDMs) but also the consideration of species traits (Floury et al., 2017; Jonsson & Jonsson, 2009; MacLean & Beissinger, 2017). In this study, we assessed climate change impacts by combining predictions of SDMs for three different dispersal scenarios (“no dispersal,” “free dispersal,” “restricted dispersal”) with thermal performance curves for three life stages (adults, juveniles, eggs) of the salmonid species *Salmo trutta* and *Salmo salar*. Thermal performance curves (TPCs) allowed the detailed investigation of performances for different timeframes, that is, monthly, seasonally, and yearly, and thus the identification of periods with potentially higher vulnerability in the future (Deutsch et al., 2008).

Monthly performance analyses showed in general higher future performances for eggs of both studied species with a temporal shift of the peak performance in the northern hemisphere from October to November. Previous studies have stated that spawning times could change quickly under new environmental settings (Carlson & Seamons, 2008) and Jonsson and Jonsson (2009) have even argued that the time of spawning could be delayed under future conditions, being in accordance with the shift of the peak performance identified in our results. For adults and juveniles of *Salmo trutta* and *Salmo salar*, varying monthly performance changes were observed, implying different reactions of the species to different time periods. For example, there was also a shift of the peak performance from June to May of *Salmo trutta* juveniles in the northern hemisphere. As changes in climatic conditions for a certain life stage can substantially affect later life stages (Angilletta, 2009; Fleming et al., 1997; Jonsson & Jonsson, 1993), identifying responses of different life stages to changing environments is essential. In general, fishes as ectothermic species exhibit phenotypic plasticity and thus plastic responses to temperature variations, implying that life‐history traits besides survivorship, like fecundity or development, change accordingly (Dawson et al., 2011; Schulte et al., 2011; Scranton & Amarasekare, 2017). Therefore, analyses of species responses on a monthly basis for different life stages enable a more detailed identification of delayed or shifted species’ traits.

Mean performances increased for the spawning seasons as well as winter, spring, and autumn in 2050s for both hemispheres and all life stages of *Salmo trutta*. Rising temperatures affected adults and juveniles of *Salmo trutta* especially during the summer season (June–August) of the northern hemisphere, where performance decreases were observable. In summer, the inclusion of different dispersal scenarios outlined the importance of dispersal possibilities in order to escape the increasing temperatures and to reach habitats where higher performances may be possible. In regard to increases in heat events (Field et al., 2014; Scranton & Amarasekare, 2017), movement through the hydrological network will be a major factor influencing survival. For the summer season of the southern hemisphere (December–February) similar statements could be made, as the “no dispersal” scenario indicated a markedly lower future mean performance than the remaining scenarios. Further on, considering that the reproduction phase and its temperature requirements is a critical bottleneck in the life cycle of fish (Dahlke et al., 2020), the ability of dispersal is even more important for the life stage eggs because higher performances were present under dispersal scenarios accounting for species movements and thus spawning in new habitats. Major differences among the seasonal mean performances of the three dispersal scenarios were absent for *Salmo salar*, which can be ascribed to the lower numbers of presences for each scenario and the catchment‐scale used for analysis. However, performances seemed to increase for every season for *Salmo salar* in the northern hemisphere, whereas for the southern hemisphere no analyses could be executed due to low numbers of occurrences. In regard of the geographical distribution of the seasonal performances for both species, the “restricted dispersal” scenarios, which accounted for dams as dispersal barriers, highlighted the negative influences of dams on species distributions, since areas with high thermal performances identified by the “free dispersal” scenario could not be reached anymore. Dams are already known to disrupt the hydrological habitat connectivity and thus aggravating climate change influences (Markovic et al., 2017).

Future distribution patterns retrieved from the SDMs under consideration of the “free” and “restricted dispersal” scenario implied a decline in suitable habitat for the 2050s and northward and southward shifts for both species in the northern and southern hemisphere, respectively. For example, the “free” and “restricted dispersal” scenario predicted a decline by −20% and by −35%, respectively, for *Salmo trutta* compared with the current global distribution range. Projections of previous studies finding a reduction in the number of suitable habitats for brown trout *Salmo trutta* are thus confirmed by our results (Wenger et al., 2011). In addition, northward movements are already projected for *Salmo salar* and highlighting that increases in water temperature may influence species traits, which can lead ultimately to extinctions for southern ranges in the northern hemisphere (Jonsson & Jonsson, 2009). With the summer season being most critical to species in regard to performance in the southern ranges, conservation actions need to focus on providing access to northern habitats within this season to prevent severe impacts for southern populations. Future higher annual mean performances in the northern USA and central to northern Europe and lower performances for the southern distribution ranges for all scenarios and life stages underline the shifts based on the studied biotic factor. However, there have to be further investigations carried out to analyze whether the inclusion of other species’ traits follows the same patterns (MacLean & Beissinger, 2017).

Few studies have tested the combination of species functional traits and model‐based predictions for species (Elmendorf & Moore, 2008; Nagaraju et al., 2013; Thuiller et al., 2010; Wittmann et al., 2016). However, these studies have found existing correlations between the model outputs and species traits. In particular, Wittmann et al. (2016) added the first fish example to the correlation analysis, finding a positive relationship (*r* = .5) between modeled habitat suitability and growth rates for the Grass Carp (*Ctenopharyngodon idella*). In this study, we investigated the relationship between modeled probabilities of occurrence and the annual mean performances for the different life stages of the salmonids. For *Salmo trutta*, we have found significant positive relationships for all three life stages. Bravais–Pearson correlation coefficients were around *r* = .40 for adults and juveniles and around *r* = .35 for eggs. For *Salmo salar*, only for eggs a significant positive relationship (*r* = .28) was found. Less studied occurrences of *Salmo salar* (*n* = 199) compared with *Salmo trutta* (*n* = 730) at the analyzed catchment scale may have impaired the found relationships. Overall, these results add further answers to the question of whether species distribution models are somehow able to account for traits through calibrations with abiotic environmental data. Further studies confronting SDMs with performance data are necessary for deriving a profound answer to this question.

Although we have included species traits for assessing climate change impacts by using thermal performance curves (TPCs), such an analysis comes with limitations and should be viewed in the context of this study and the used model for deriving thermal performance curves. TPCs were based on experimentally observed data for different life stages not representing true settings in the field. Acclimation processes in laboratory experiments can substantially modify observed thermal limits and thus the shape of TPCs (Angilletta, 2009). Furthermore, resource limitations in the field can alter the temperature performance relationship as well as the interaction of temperature with a variety of biotic and abiotic factors (Angilletta, 2009; Childress & Letcher, 2017; Schulte et al., 2011). Martin et al. (2016) have found that laboratory data can significantly underestimate field‐derived thermal mortality. In addition, due to both genetic and nongenetic reasons single individuals in populations may have significantly differing thermal properties (Kingsolver et al., 2011). As such, the preferred temperature (*T*_opt_) and thermal tolerance may vary from species to species, between populations and even individuals and therefore lead to differences in the shape of thermal performance curves (Angilletta, 2009). Intraspecific variations in thermal tolerance might influence the width of TPCs and need to be considered when estimating climate change impacts for different geographical locations (Fangue et al., 2006). Despite these differences, other previous studies have found no significant relationships between a species’ functional trait or performance and the thermal conditions of different populations (Angilletta, 2009; Elliott & Elliott, 2010; Forseth et al., 2009; Jonsson et al., 2001; Jonsson & Jonsson, 2009). However, *T*_opt_ is influenced by further factors such as the amplitude of thermal cycles and variations which species have recently been exposed to or levels of dissolved oxygen (Jobling, 1981; Johnson & Kelsch, 1998). Especially for eggs, a strong relationship between oxygen limitation and thermal tolerance of fish embryos was identified (Martin et al., 2016). Moreover, for this life stage varying response types might be possible (see Tsoukali et al., 2016). The found thermal limits CT_min_ and CT_max_ are also not necessarily survival limits as species may endure short‐term exposures to temperatures beyond these limits (Sinclair et al., 2016). Brief exposure to such temperatures can even cause greater tolerance to temperature extremes, which is called hardening (Angilletta, 2009). Consequently, the duration of the exposure to critical temperatures additionally influences the performance, where the performance usually decreases with increasing exposure time (Sinclair et al., 2016). Shifting from static to dynamic TPCs, which incorporate a time component, would enable more comprehensive and realistic studies of climate change impacts (Schulte et al., 2011; Woodin et al., 2013). However, current data availability restricts such analyses.

In summary, future temperature changes will influence the performance of each life stage of the studied fish species differently according to the analyzed timeframes. Dispersal possibilities will become more important for fish distributions in order to escape warming and reach areas where performance can increase. Dams as dispersal barriers disrupt catchment connectivity and will impede movement to suitable habitats linked to high performance values. Thus, we suggest that conservation management should incorporate a time component enabling the mitigation of severe climate change effects in periods where performances of species might drop critically. Additionally, catchments where dispersal barriers are present and prohibiting movement to places where higher performances could be possible should be reconsidered in further conservation planning.

## CONFLICT OF INTEREST

The authors declare no conflict of interest.

## AUTHOR CONTRIBUTIONS

**Oskar Kärcher:** Conceptualization (lead); Data curation (lead); Formal analysis (lead); Methodology (equal); Validation (lead); Visualization (lead); Writing‐original draft (lead); Writing‐review & editing (equal). **Martina Flörke:** Data curation (lead); Resources (equal); Writing‐review & editing (equal). **Danijela Markovic:** Conceptualization (supporting); Data curation (lead); Formal analysis (supporting); Funding acquisition (lead); Methodology (equal); Project administration (lead); Supervision (lead); Writing‐original draft (supporting); Writing‐review & editing (equal).

## Supporting information

Appendix S1

Appendix S2

## Data Availability

The full data set used for the modeling of the considered fish species distributions is provided as a Supplementary file.

## References

[ece37731-bib-0001] Angert, A. L. , Sheth, S. N. , & Paul, J. R. (2011). Incorporating population‐level variation in thermal performance into predictions of geographic range shifts. Integrative and Comparative Biology, 51, 733–750. 10.1093/icb/icr048 21705795

[ece37731-bib-0002] Angilletta, M. J. (2006). Estimating and comparing thermal performance curves. Journal of Thermal Biology, 31, 541–545. 10.1016/j.jtherbio.2006.06.002

[ece37731-bib-0003] Angilletta, M. (2009). Thermal adaptation: A theoretical and empirical synthesis. Oxford University Press.

[ece37731-bib-0004] Angilletta, M. J. , Niewiarowski, P. H. , & Navas, C. A. (2002). The evolution of thermal physiology in ectotherms. Journal of Thermal Biology, 27, 249–268. 10.1016/S0306-4565(01)00094-8

[ece37731-bib-0005] Beck, H. E. , van Dijk, A. I. J. M. , de Roo, A. , Dutra, E. , Fink, G. , Orth, R. , & Schellekens, J. (2017). Global evaluation of runoff from 10 state‐of‐the‐art hydrological models. Hydrology and Earth System Sciences, 21, 2881–2903. 10.5194/hess-21-2881-2017

[ece37731-bib-0006] Beitinger, T. L. , Bennett, W. A. , & McCauley, R. W. (2000). Temperature tolerances of North American freshwater species exposed to dynamic changes in temperature. Environmental Biology of Fishes, 58, 237–275.

[ece37731-bib-0007] Bergstra, J. , & Bengio, Y. (2012). Random search for hyper‐parameter optimization. Journal of Machine Learning Research, 13, 2817–3305.

[ece37731-bib-0008] Bishop, C. M. (1995). Neural networks for pattern recognition. Oxford University Press Inc.

[ece37731-bib-0009] Brauman, K. A. , Richter, B. D. , Postel, S. , Malsy, M. , & Flörke, M. (2016). Water depletion: An improved metric for incorporating seasonal and dry‐year water scarcity into water risk assessments. Elementa: Science of Anthropocene, 4, 83.

[ece37731-bib-0010] Breiman, L. (2001). Statistical modelling: The two cultures. Statistical Science, 16, 199–215.

[ece37731-bib-0011] Brett, J. R. (1952). Temperature tolerance in young Pacific salmon genus Oncorhynchus. Journal of the Fisheries Research Board of Canada, 9, 265–323. 10.1139/f52-016

[ece37731-bib-0012] Campbell, J. S. (1977). Spawning characteristics of brown trout and sea trout Salmo trutta L. in Kirk Burn, River Tweed, Scotland. Journal of Fish Biology, 11, 217–229. 10.1111/j.1095-8649.1977.tb04115.x

[ece37731-bib-0013] Carlson, S. M. , & Seamons, T. R. (2008). A review of quantitative genetic components of fitness in salmonids: Implications for adaptation to future change. Evolutionary Applications, 1, 222–238. 10.1111/j.1752-4571.2008.00025.x 25567628 PMC3352437

[ece37731-bib-0014] Childress, E. S. , & Letcher, B. H. (2017). Estimating thermal performance curves from repeated field observations. Ecology, 98, 1377–1387. 10.1002/ecy.1801 28273358

[ece37731-bib-0015] Comte, L. , Murienne, J. , & Grenouillet, G. (2014). Species traits and phylogenetic conservatism of climate‐induced range shifts in stream fishes. Nature Communications, 5, 5023. 10.1038/ncomms6053 PMC5898465 25248802

[ece37731-bib-0016] Dahlke, F. T. , Wohlrab, S. , Butzin, M. , & Pörtner, H.‐O. (2020). Thermal bottlenecks in the life cycle define climate vulnerability of fish. Science, 369, 65–70. 10.1126/science.aaz3658 32631888

[ece37731-bib-0017] Dawson, T. P. , Jackson, S. T. , House, J. I. , Prentice, I. C. , & Mace, G. M. (2011). Beyond predictions: Biodiversity conservation in a changing climate. Science, 332, 53–58. 10.1126/science.1200303 21454781

[ece37731-bib-0018] DEFRA (2013). Catchment Based Approach: Improving the quality of our water environment. Department for Environment, Food & Rural Affairs. Policy paper avaialble at: https://www.gov.uk/government/publications/catchment‐based‐approach‐improving‐the‐quality‐of‐our‐water‐environment

[ece37731-bib-0019] Dell, A. , Pawar, S. , & Savage, V. M. (2011). Systematic variation in the temperature dependence of physiological and ecological traits. Proceedings of the National Academy of Sciences of the United States of America, 108, 10591–10596. 10.1073/pnas.1015178108 21606358 PMC3127911

[ece37731-bib-0020] Deutsch, C. A. , Tewksbury, J. J. , Huey, R. B. , Sheldon, K. S. , Ghalambor, C. K. , Haak, D. C. , & Martin, P. R. (2008). Impacts of climate warming on terrestrial ectotherms across latitude. Proceedings of the National Academy of Sciences of the United States of America, 105, 6668–6672. 10.1073/pnas.0709472105 18458348 PMC2373333

[ece37731-bib-0021] Dormann, C. F. , Purschke, O. , Márquez, J. R. G. , Lautenbach, S. , & Schröder, B. (2008). Components of uncertainty in species distribution analysis: A case study of the great grey shrike. Ecology, 89, 3371–3386. 10.1890/07-1772.1 19137944

[ece37731-bib-0022] Duda, R. , Hart, P. , & Stork, D. (2001). Pattern classification. Wiley‐Interscience.

[ece37731-bib-0023] Dudgeon, D. , Arthington, A. H. , Gessner, M. O. , Kawabata, Z. , Knowler, D. J. , Lévêque, C. , Naiman, R. J. , Prieur‐Richard, A. H. , Soto, D. , Stiassny, M. L. , & Sullivan, C. A. (2006). Freshwater biodiversity: importance, threats, status and conservation challenges. Biological Reviews, 81, 163–182. 10.1017/S1464793105006950 16336747

[ece37731-bib-0024] EEA (European Environment Agency) : Corine Land Cover 2000 (2004). Mapping a decade of change Document Actions, Tech. Rep. Brochure No 4/2004.

[ece37731-bib-0025] Eisner, S. (2016). Comprehensive evaluation of the WaterGAP3 model across climatic, physiographic, and anthropogenic gradients. Dissertation, KOBRA Dokumentenserver, University of Kassel, Germany. http://nbn‐resolving.de/urn:nbn:de:hebis:34‐2016031450014

[ece37731-bib-0026] Eisner, S. , Flörke, M. , Chamorro, A. , Daggupati, P. , Donnelly, C. , Huang, J. , & Krysanova, V. (2017). An ensemble analysis of climate change impacts on streamflow seasonality across 11 large river basins. Climatic Change, 141, 401–417. 10.1007/s10584-016-1844-5

[ece37731-bib-0027] El‐Gabbas, A. , & Dormann, C. F. (2017). Improved species‐occurrence predictions in data‐poor regions: Using large‐scale data and bias correction with down‐weighted Poisson regression and Maxent. Ecography, 41, 1161–1172. 10.1111/ecog.03149

[ece37731-bib-0028] Elith, J. , Leathwick, J. R. , & Hastie, T. (2008). A Working Guide to Boosted Regression Trees. Journal of Animal Ecology, 77, 802–813. 10.1111/j.1365-2656.2008.01390.x 18397250

[ece37731-bib-0029] Elliott, J. M. (1994). Quantitative ecology and the brown trout. Oxford Series in Ecology and Evolution. Oxford University Press.

[ece37731-bib-0030] Elliott, J. M. , & Elliott, J. A. (2010). Temperature requirements of Atlantic salmon Salmo salar, brown trout Salmo trutta and Arctic charr Salvelinus alpinus: predicting the effects of climate change. Journal of Fish Biology, 77, 1793–1817. 10.1111/j.1095-8649.2010.02762.x 21078091

[ece37731-bib-0031] Elmendorf, S. , & Moore, K. (2008). Use of communitycomposition data to predict the fecundity and abundance of species. Conservation Biology, 22, 1523–1532. 10.1111/j.1523-1739.2008.01051.x 18847440

[ece37731-bib-0032] Fagan, W. F. (2002). Connectivity, fragmentation, and extinction risk in dendritic metapopulations. Ecology, 83, 3243–3249.

[ece37731-bib-0033] Fangue, N. A. , Hofmeister, M. , & Schulte, P. M. (2006). Intraspecific variation in thermal tolerance and heat shock protein gene expression in common killifish, *Fundulus heteroclitus* . Journal of Experimental Biology, 209, 2859–2872. 10.1242/jeb.02260 16857869

[ece37731-bib-0034] Field, C. , Barros, V. , Dokken, D. J. , Mach, K. J. , Mastrandrea, M. D. , Bilir, T. E. , Chatterjee, M. , Ebi, K. L. , Estrada, Y. O. , Genova, R. C. , Girma, B. , Kissel, E. S. , Levy, A. N. , MacCracken, S. , Mastrandrea, P. R. , & White, L. L. (2014). Climate Change 2014 ‐ Impacts, adaptation and vulnerability: Part A: Global and sectoral aspects. Cambridge University Press.

[ece37731-bib-0035] Fielding, A. H. , & Bell, J. F. (1997). A review of methods for the assessment of prediction errors in conservation presence/ absence models. Environmental Conservation, 24, 38–49. 10.1017/S0376892997000088

[ece37731-bib-0036] Fleming, I. A. , Lamberg, A. , & Jonsson, B. (1997). Effects of early experience on the reproductive performance of Atlantic salmon. Behavioural Ecology, 8, 470–480. 10.1093/beheco/8.5.470

[ece37731-bib-0037] Floury, M. , Usseglio‐Polatera, P. , Delattre, C. , & Souchon, Y. (2017). Assessing long‐term effects of multiple, potentially confounded drivers in ecosystems from species traits. Global Change Biology, 23, 2297–2307. 10.1111/gcb.13575 27873443

[ece37731-bib-0038] Forseth, T. , Larsson, S. , Jensen, A. J. , Jonsson, B. , Näslund, I. , & Berglund, I. (2009). Thermal performance of juvenile brown trout, *Salmo trutta* L.: no support for thermal adaptation hypotheses. Journal of Fish Biology, 74, 133–149. 20735529 10.1111/j.1095-8649.2008.02119.x

[ece37731-bib-0039] Fourcade, Y. (2016). Comparing species distributions modeled from occurrence data and from expert‐based range maps. Implication for predicting range shifts with climate change. Ecological Informatics, 36, 8–14.

[ece37731-bib-0040] Frazier, M. R. , Huey, R. B. , & Berrigan, D. (2006). Thermodynamics constrains the evolution of insect population growth rates: “Warmer is better”. American Naturalist, 168, 512–520. 10.1086/506977 17004222

[ece37731-bib-0041] Friedman, J. H. (1991). Multivariate adaptive regression splines (with discussion). The Annals of Statistics, 19, 1–141.

[ece37731-bib-0042] Friedman, J. H. , Hastie, T. , & Tibshirani, R. (2010). Regularization paths for generalized linear models via coordinate descent. Journal of Statistical Software, 33, 1. 10.18637/jss.v033.i01 20808728 PMC2929880

[ece37731-bib-0043] Grenouillet, G. , Buisson, L. , Casajus, N. , & Lek, S. (2011). Ensemble modelling of species distribution: The effects of geographical and environmental ranges. Ecography, 34, 9–17. 10.1111/j.1600-0587.2010.06152.x

[ece37731-bib-0044] Guisan, A. , & Thuiller, W. (2005). Predicting species distribution: Offering more than simple habitat models. Ecology Letters, 8, 993–1009. 10.1111/j.1461-0248.2005.00792.x 34517687

[ece37731-bib-0045] Hastie, T. , & Tibshirani, R. (1986). Generalized additive models. Statistical Science, 1, 297–318. 10.1177/096228029500400302 8548102

[ece37731-bib-0046] Hastie, T. , Tibshirani, R. , & Friedman, J. (2001). The elements of statistical learning (vol. 1). Springer.

[ece37731-bib-0047] He, B. , Oki, T. , Sun, F. , Komori, D. , Kanae, S. , Wang, Y. , Kim, H. , & Yamazaki, D. (2011). Estimating monthly total nitrogen concentration in streams by using artificial neural network. Journal of Environmental Economics and Management, 92, 172–177. 10.1016/j.jenvman.2010.09.014 20870340

[ece37731-bib-0048] Hempel, S. , Frieler, K. , Warszawski, L. , Schewe, J. , & Piontek, F. (2013). A trend‐preserving bias correction – the ISI‐MIP approach. Earth System Dynamics, 4, 49–92.

[ece37731-bib-0049] Hereford, J. (2009). A quantitative survey of local adaptation and fitness trade‐offs. American Naturalist, 173, 579–588. 10.1086/597611 19272016

[ece37731-bib-0050] Hijmans, R. J. , Phillips, S. , Leathwick, J. , & Elith, J. (2017). Package ‘dismo’, R package version 1.1‐1. http://cran.r‐project.org/web/packages/dismo/index.html

[ece37731-bib-0051] Hochochka, P. W. , & Somero, G. N. (2002). Biochemical adaptation. Oxford University Press.

[ece37731-bib-0052] Huey, R. , & Kingsolver, J. G. (1989). Evolution of thermal sensitivity of ectotherm performance. Trends in Ecology & Evolution, 4, 131–135. 10.1016/0169-5347(89)90211-5 21227334

[ece37731-bib-0053] Huey, R. B. , & Stevenson, R. D. (1979). Integrating thermal physiology and ecology of ectotherms: a discussion of approaches. American Zoologist, 19(1), 357–366. 10.1093/icb/19.1.357

[ece37731-bib-0054] Jain, A. K. , Mao, J. , & Mohiuddin, K. M. (1996). Artificial neural networks: A tutorial. Computer, 29(3), 31–44. 10.1109/2.485891

[ece37731-bib-0055] Jatteau, P. , Drouineau, H. , Charles, K. , Carry, L. , Lange, F. , & Lambert, P. (2017). Thermal tolerance of allis shad (Alosa alosa) embryos and larvae: Modeling and potential applications. Aquatic Living Resources, 30, 2.

[ece37731-bib-0056] Jimenez‐Valverde, A. , & Lobo, J. M. (2007). Threshold criteria for conversion of probability of species presence to either‐or presence‐absence. Acta Oecologica, 31(3), 361–369. 10.1016/j.actao.2007.02.001

[ece37731-bib-0057] Jobling, M. (1981). Temperature tolerance and the final preferendum – rapid methods for the assessment of optimum growth temperatures. Journal of Fish Biology, 19, 439–455. 10.1111/j.1095-8649.1981.tb05847.x

[ece37731-bib-0058] Johnson, J. A. , & Kelsch, S. W. (1998). Effects of evolutionary thermal environment on temperature‐preference relationships in fishes. Environmental Biology of Fishes, 53, 447–458. 10.1023/A:1007425215669

[ece37731-bib-0059] Jonsson, B. , Forseth, T. , Jensen, A. J. , & Naesje, T. F. (2001). Thermal performance of juvenile Atlantic salmon, Salmo salar L. Functional Ecology, 15, 701–711.

[ece37731-bib-0060] Jonsson, B. , & Jonsson, N. (1993). Partial migration: Niche shift versus sexual maturation in fishes. Reviews in Fish Biology and Fisheries, 3, 348–365. 10.1007/BF00043384

[ece37731-bib-0061] Jonsson, B. , & Jonsson, N. (2009). A review of the likely effects of climate change on anadromous Atlantic salmon Salmo salar and brown trout Salmo trutta, with particular reference to water temperature and flow. Journal of Fish Biology, 75, 2381–2447. 20738500 10.1111/j.1095-8649.2009.02380.x

[ece37731-bib-0062] Jonsson, B. , & Jonsson, N. (2011). Ecology of Atlantic Salmon and Brown Trout: Habitat as a template for life histories. Springer Science & Business Media.

[ece37731-bib-0063] Jonsson, B. , & Jonsson, N. (2014). Early environments affect later performances in fishes. Journal of Fish Biology, 85, 155–188. 10.1111/jfb.12432 24961386

[ece37731-bib-0064] Jonsson, B. , & L’Abée‐Lund, J. H. (1993). Latitudinal clines in life history variables of anadromous brown trout in Europe. Journal of Fish Biology, 43, 1–16. 10.1111/j.1095-8649.1993.tb01175.x

[ece37731-bib-0065] Kingsolver, J. G. , Woods, H. A. , Buckley, L. B. , Potter, K. A. , MacLean, H. J. , & Higgins, J. K. (2011). Complex life cycles and the responses of insects to climate change. Integrative and Comparative Biology, 51, 719–732. 10.1093/icb/icr015 21724617

[ece37731-bib-0066] Krenek, S. , Berendonk, T. U. , & Petzoldt, T. (2011). Thermal performance curves of Paramecium caudatum: A model selection approach. European Journal of Protistology, 47, 124–137. 10.1016/j.ejop.2010.12.001 21277756

[ece37731-bib-0067] Kümmerlen, M. , Reichert, P. , Siber, R. , & Schuwirth, N. (2019). Ecological assessment of river networks: From reach to catchment scale. Science of Total Environment, 1613–1627. 10.1016/j.scitotenv.2018.09.019 30308847

[ece37731-bib-0068] Lee, K. Y. , Chung, N. , & Hwang, S. (2016). Application of an artificial neural network (ANN) model for predicting mosquito abundances in urban areas. Ecological Informatics, 36, 172–180. 10.1016/j.ecoinf.2015.08.011

[ece37731-bib-0069] Lehner, B. , Liermann, C. R. , Revenga, C. , Vörösmarty, C. , Fekete, B. , Crouzet, P. , & Wisser, D. (2011). High‐resolution mapping of the world’s reservoirs and dams for sustainable river‐flow management. Frontiers in Ecology and the Environment, 9, 494–502. 10.1890/100125

[ece37731-bib-0070] Lévêque, C. , Oberdorff, T. , Paugy, D. , Stiassny, M. L. J. , & Tedesco, P. A. (2008). Global diversity of fish (Pisces) in freshwater. Hydrobiologia, 595, 545–567. 10.1007/s10750-007-9034-0

[ece37731-bib-0071] Li, X. , & Wang, Y. (2013). Applying various algorithms for species distribution modelling. Integrative Zoology, 8, 124–135. 10.1111/1749-4877.12000 23731809

[ece37731-bib-0072] MacLean, S. , & Beissinger, S. R. (2017). Species' traits as predictors of range shifts under contemporary climate change: A review and meta‐analysis. Global Change Biology, 23, 4094–4105. 10.1111/gcb.13736 28449200

[ece37731-bib-0073] Markovic, D. , Carrizo, S. F. , Kärcher, O. , Walz, A. , & David, J. N. W. (2017). Vulnerability of European freshwater catchments to climate change. Global Change Biology, 23, 3567–3580. 10.1111/gcb.13657 28186382

[ece37731-bib-0074] Marmion, M. , Parviainen, M. , Luoto, M. , Heikkinen, R. K. , & Thuiller, W. (2009). Evaluation of consensus methods in predictive species distribution modelling. Diversity and Distributions, 15, 59–69. 10.1111/j.1472-4642.2008.00491.x

[ece37731-bib-0075] Martin, B. T. , Pike, A. , John, S. N. , Hamda, N. , Roberts, J. , Lindley, S. T. , & Danner, E. M. (2016). Phenomenological vs. biophysical models of thermal stress in aquatic eggs. Ecology Letters, 20, 50–59. 10.1111/ele.12705 27891770

[ece37731-bib-0076] Merow, C. , Allen, J. M. , Aiello‐Lammens, M. , & Silander, J. A. (2016). Improving niche and range estimates with Maxent and point process models by integrating spatially explicit information. Global Ecology and Biogeography, 25, 1022–1036. 10.1111/geb.12453

[ece37731-bib-0077] Milborrow, S. (2018). Derived from mda:mars by T. Hastie and R. Tibshirani. Earth: Multivariate Adaptive Regression Splines, R package version 4.4.7. http://www.milbo.users.sonic.net/earth

[ece37731-bib-0078] Nagaraju, S. K. , Gudasalamani, R. , Barve, N. , Ghazoul, J. , Narayanagowda, G. K. , & Ramanan, U. S. (2013). Do ecological niche model predictions reflect the adaptive landscape of species? A test using *Myristica malabarica* Lam., an endemic tree in the Western Ghats, India. PLoS One, 8, e82066. 24312402 10.1371/journal.pone.0082066 PMC3843714

[ece37731-bib-0079] O'Donnell, M. J. , Regish, A. M. , McCormick, S. D. , & Letcher, B. H. (2020). How repeatable is CT_max_ within individual brook trout over short‐ and long‐time intervals? Journal of Thermal Biology, 89. 10.1016/j.jtherbio.2020.102559 32364992

[ece37731-bib-0080] Östergren, J. , & Rivinoja, P. (2008). Overwintering and downstream migration of Sea sea trout (*Salmo trutta* L.) kelts under regulated flows – northern Sweden. River Research and Applications, 24, 551–563.

[ece37731-bib-0081] Ovidio, M. , & Philippart, J. C. (2002). The impact of small physical obstacles on upstream movements of six species of fish. In E. B. Thorstad , I. A. Fleming , & T. F. Næsje (Eds.), Aquatic Telemetry. Developments in hydrobiology (vol. 165). Springer.

[ece37731-bib-0082] Pachauri, R. K. , & Mayer, L. (Eds.) (2015). Climate change 2014. Synthesis report (151 p). Intergovernmental Panel on Climate Change.

[ece37731-bib-0083] Pankhurst, N. W. , & King, H. R. (2010). Temperature and salmonid reproduction: implications for aquaculture. Journal of Fish Biology, 76, 69–85. 10.1111/j.1095-8649.2009.02484.x 20738700

[ece37731-bib-0084] Phillips, S. J. , Anderson, R. P. , & Schapire, R. E. (2006). Maximum entropy modeling of species geographic distributions. Ecological Modelling, 190, 231–259. 10.1016/j.ecolmodel.2005.03.026

[ece37731-bib-0085] Phillips, S. J. , & Dudik, M. (2008). Modeling of species distributions with Maxent: New extensions and a comprehensive evaluation. Ecography, 31, 161–175. 10.1111/j.0906-7590.2008.5203.x

[ece37731-bib-0086] Punzet, M. , Voß, F. , Voß, A. , Kynast, E. , & Bärlund, I. (2012). A global approach to assess the potential impact of climate change on stream water temperatures and related in‐stream first‐order decay rates. Journal of Hydrometeorology, 13, 1052–1065. 10.1175/JHM-D-11-0138.1

[ece37731-bib-0087] R Development Core Team (2018). R: A language and environment for statistical computing. R Foundation for Statistical Computing.

[ece37731-bib-0088] Riahi, K. , Rao, S. , Krey, V. , Cho, C. , Chirkov, V. , Fischer, G. , & Rafaj, P. (2011). RCP8.5 – A scenario of comparatively high greenhouse gas emissions. Climatic Change, 109, 33–57. 10.1007/s10584-011-0149-y

[ece37731-bib-0089] Rocchini, D. , Hortal, J. , Lengyel, S. , Lobo, J. M. , Jimenez‐Valverde, A. , Ricotta, C. , Bacaro, G. , & Chiarucci, A. (2011). Accounting for uncertainty when mapping species distributions: The need for maps of ignorance. Progress in Physical Geography, 35, 211–226. 10.1177/0309133311399491

[ece37731-bib-0090] Rome, L. C. , Stevens, E. D. , & John‐Alder, H. B. (1992). The influence of temperature and thermal acclimation on physiological function. In M. E. Feder , & W. W. Burggrem (Eds.), Environmental physiology of the Amphibians (pp. 183–205). The University of Chicago Press.

[ece37731-bib-0091] Schneider, C. , Flörke, M. , De Stefano, L. , & Petersen‐Perlman, J. D. (2017). Hydrological threats for riparian wetlands of international importance – a global quantitative and qualitative analysis. Hydrology and Earth System Sciences, 21, 2799–2815.

[ece37731-bib-0092] Schulte, P. M. , Healy, T. M. , & Fangue, N. A. (2011). Thermal performance curves, phenotypic plasticity, and the time scales of temperature exposure. Integrative and Comparative Biology, 51, 691–702. 10.1093/icb/icr097 21841184

[ece37731-bib-0093] Scranton, K. , & Amarasekare, P. (2017). Predicting phenological shifts in a changing climate. Proceedings of the National Academy of Sciences of the United States of America, 114, 13212–13217. 10.1073/pnas.1711221114 29180401 PMC5740618

[ece37731-bib-0094] Sinclair, B. J. , Marshall, K. E. , Sewell, M. A. , Levesque, D. L. , Willett, C. S. , Slotsbo, S. , & Huey, R. B. (2016). Can we predict ectotherm responses to climate change using thermal performance curves and body temperatures? Ecology Letters, 19(11), 1372–1385. 10.1111/ele.12686 27667778

[ece37731-bib-0095] Souchon, Y. , & Tissot, L. (2012). Synthesis of thermal tolerances of the common freshwater fish species in large Western Europe rivers. Knowledge and Management of Aquatic Ecosystems, 405, 3. 10.1051/kmae/2012008

[ece37731-bib-0096] The H2O.ai team (2018). h2o: R Interface for H2O. R package version 3.20.0.8. https://cran.r‐project.org/web/packages/h2o/h2o.pdf

[ece37731-bib-0097] Thuiller, W. , Albert, C. H. , Dubuis, A. , Randin, C. , & Guisan, A. (2010). Variation in habitat suitability does not always relate to variation in species’ plant functional traits. Biology Letters, 6, 120–123. 10.1098/rsbl.2009.0669 19793738 PMC2817270

[ece37731-bib-0098] Thuiller, W. , Lafourcade, B. , Engler, R. , & Araujo, M. B. (2009). BIOMOD–a platform for ensemble forecasting of species distributions. Ecography, 32, 369–373. 10.1111/j.1600-0587.2008.05742.x

[ece37731-bib-0099] Tsoukali, S. , Visser, A. , & MacKenzie, B. (2016). Functional responses of North Atlantic fish eggs to increasing temperature. Marine Ecology Progress Series, 555, 151–165. 10.3354/meps11758

[ece37731-bib-0100] USGS: Global Land Cover Characterization (GLCC) (2008). https://www.usgs.gov/centers/eros/science/usgs‐eros‐archive‐land‐cover‐products‐global‐land‐cover‐characteristics‐glcc?qt‐science_center_objects=0#qt‐science_center_objects

[ece37731-bib-0101] Vasconcelos, R. P. , Batista, M. I. , & Henriques, S. (2017). Current limitations of global conservation to protect higher vulnerability and lower resilience fish species. Scientific Reports, 7, 7702. 10.1038/s41598-017-06633-x 28794436 PMC5550462

[ece37731-bib-0102] Wenger, S. J. , Isaak, D. J. , Luce, C. H. , Neville, H. M. , Fausch, K. D. , Dunham, J. B. , Dauwalter, D. C. , Young, M. K. , Elsner, M. M. , Rieman, B. E. , Hamlet, A. F. , & Williams, J. E. (2011). Flow regime, temperature, and biotic interactions drive differential declines of trout species under climate change. Proceedings of the National Academy of Sciences of the United States of America, 108, 14175–14180. 10.1073/pnas.1103097108 21844354 PMC3161569

[ece37731-bib-0103] Wittmann, M. E. , Barnes, M. A. , Jerde, C. L. , Jones, L. A. , & Lodge, D. M. (2016). Confronting species distribution model predictions with species functional traits. Ecology and Evolution, 6, 873–879. 10.1002/ece3.1898 26941933 PMC4761765

[ece37731-bib-0104] Wood, S. N. (2011). Fast stable restricted maximum likelihood and marginal likelihood estimation of semiparametric generalized linear models. Journal of the Royal Statistical Society: Series B, 73(1), 3–36. 10.1111/j.1467-9868.2010.00749.x

[ece37731-bib-0105] Woodin, S. A. , Hilbish, T. J. , Helmuth, B. , Jones, S. J. , & Wethey, D. S. (2013). Climate change, species distribution models, and physiological performance metrics: predicting when biogeographic models are likely to fail. Ecology and Evolution, 3, 3334–3346. 10.1002/ece3.680 24223272 PMC3797481

[ece37731-bib-0106] Wootton, R. J. (1998). Ecology of teleost fishes (2nd ed.). Kluwer.

[ece37731-bib-0107] Zeiler, M. D. (2012). ADADELTA: An Adaptive Learning Rate Method. Arxiv.org. N.p. https://arxiv.org/abs/1212.5701

[ece37731-bib-0108] Zhang, W. , & Goh, A. T. (2016). Multivariate adaptive regression splines and neural network models for prediction of pile drivability. Geoscience Frontiers, 7, 45–52. 10.1016/j.gsf.2014.10.003

