# Supplementary material for: Different life stage, different risks: Thermal performance across the life cycle of Salmo trutta and Salmo salar in the face of climate change
Source: Ecol Evol. 2021 Jun 8;11(13):8941–56. doi: 10.1002/ece3.7731 (PMC8258189; doi:10.1002/ece3.7731)
Supplement: Supplementary file 1 — Appendix S1 [file ECE3-11-8941-s001.docx]

**Appendix S1**

**SUPPORTING INFORMATION**

Different life stage, different risks: thermal performance across the life cycle of *Salmo trutta* and *Salmo salar* in the face of climate change

Oskar Kärcher, Martina Flörke, & Danijela Markovic


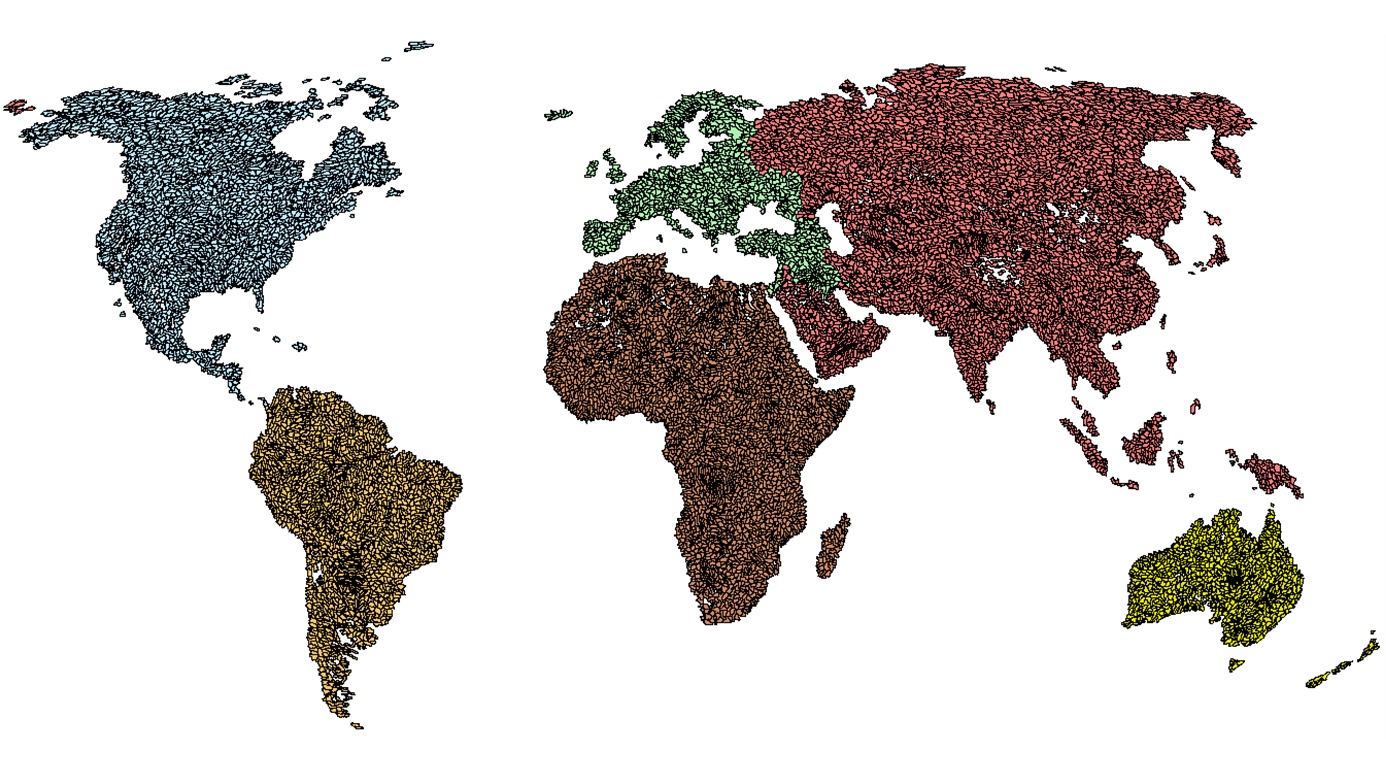


# Fig. S1 Global map of continental catchment regions. Only catchments with an area of ≥3000 km² were considered. The African region is displayed in brown colour, the Asian region in red, the Australian region in yellow, the European region in green, the North American region in blue and the South American region in orange.


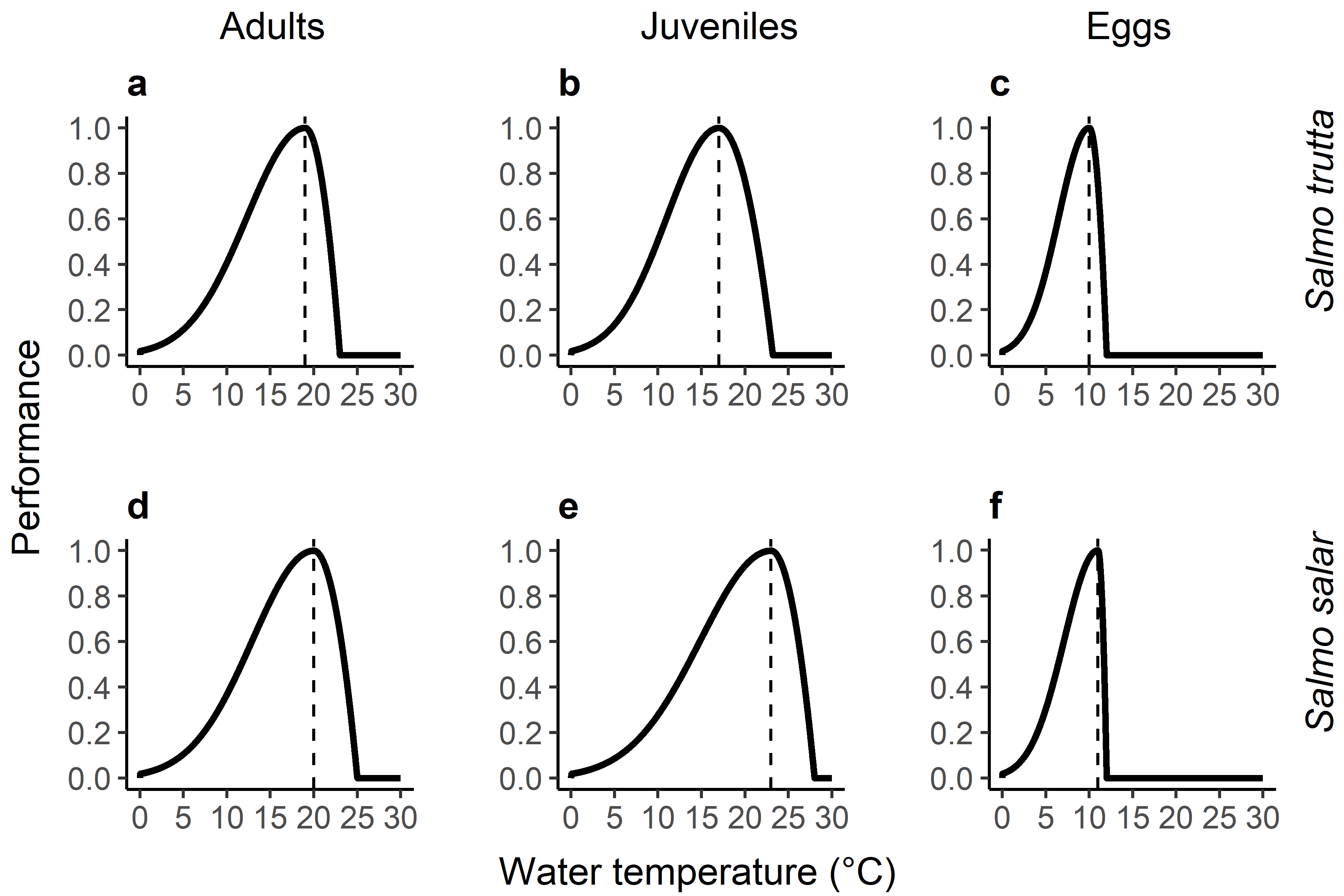


# Fig. S2 Thermal performance curves for the life stages adults, juveniles and eggs of *Salmo trutta* (a-c) and *Salmo salar* (d-f). The dotted lines mark the optimum temperatures.


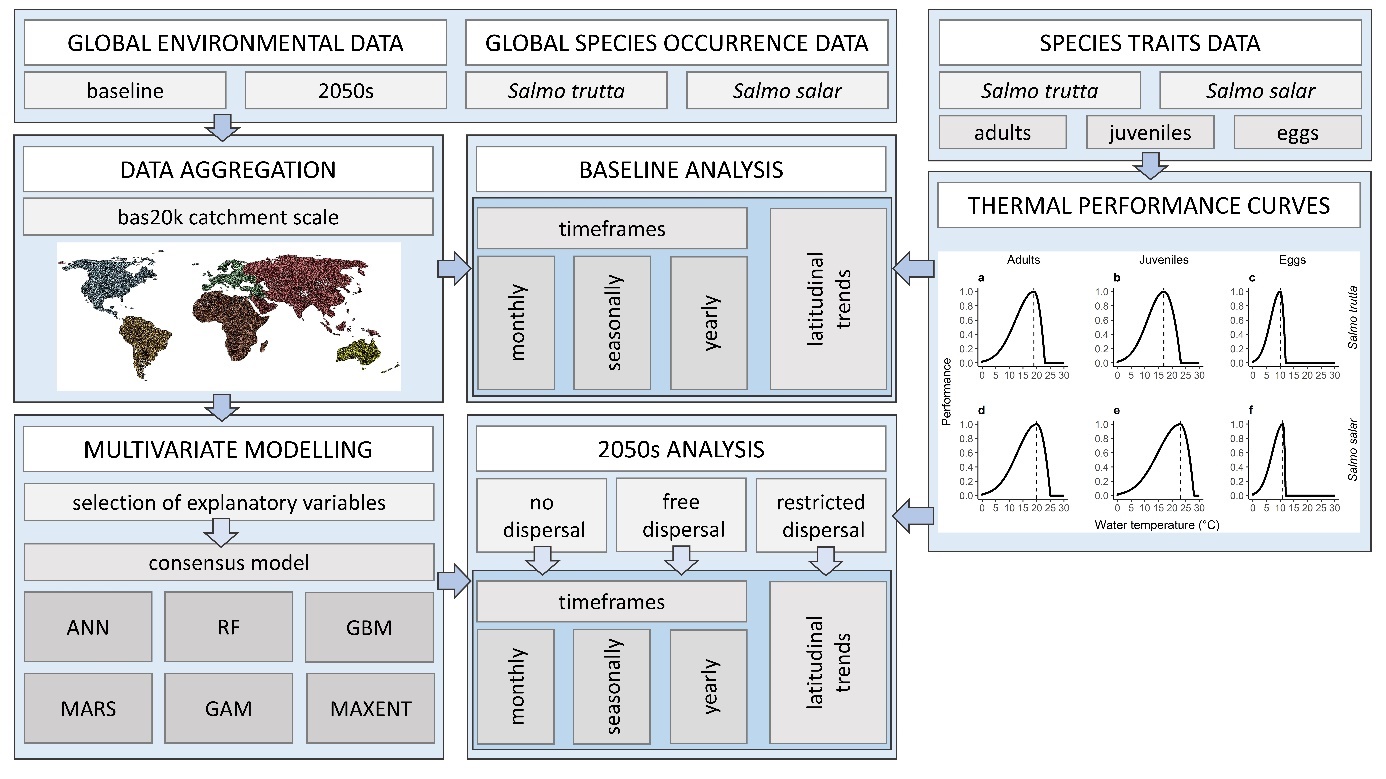


# Fig. S3 Main conceptual workflow of the study. The consensus model consisted of Artificial Neural Networks (ANN), Random Forest (RF), Gradient Boosting Machines (GBM), Multivariate Adaptive Regression Splines (MARS), Generalized Additive Models (GAM) and Maximum Entropy Method (MAXENT). Note that Elastic Net (ELNET) was excluded due to lower performance values.


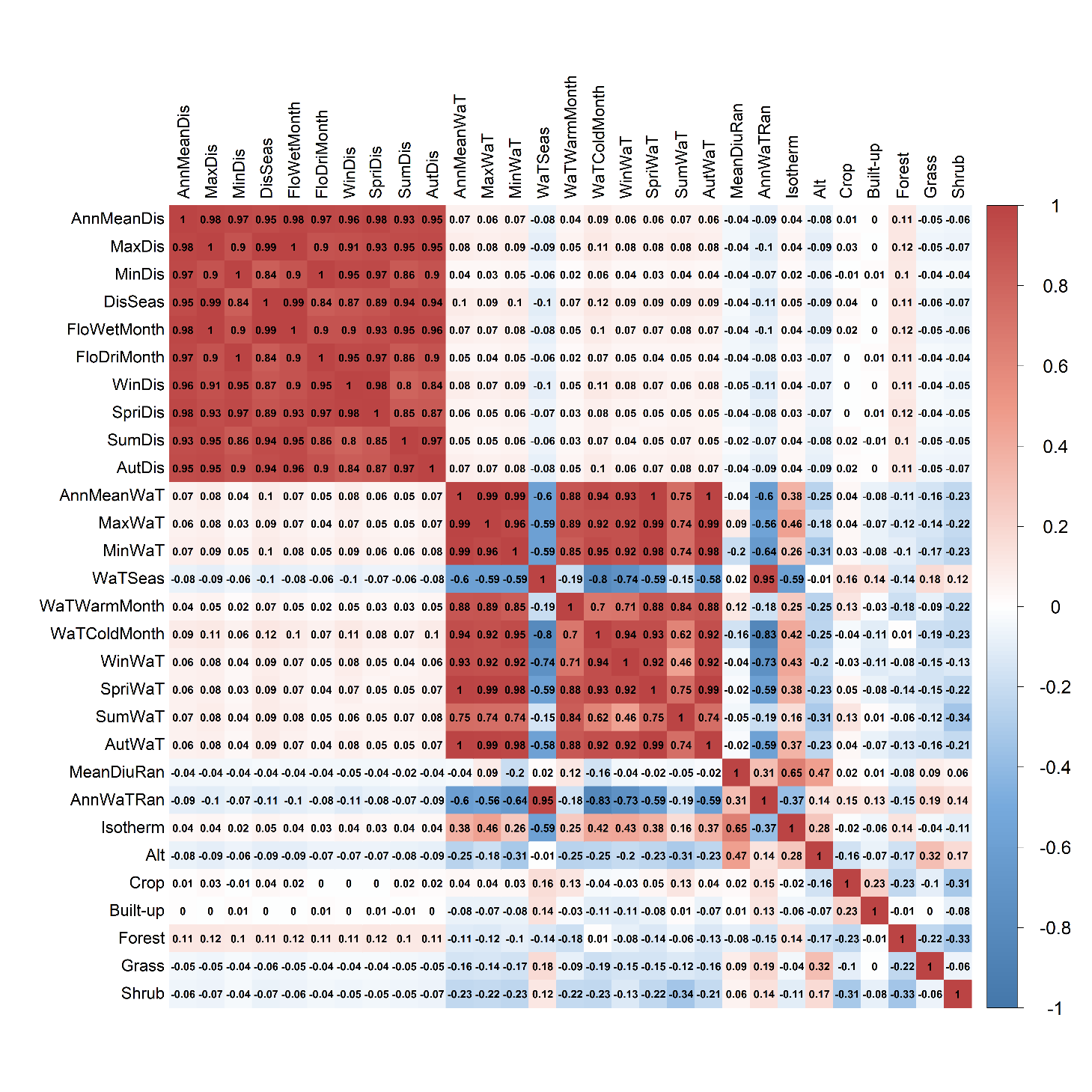


# Fig. S4 Correlation matrix indicating the value of the pairwise Bravais-Pearson correlation coefficient of the environmental predictor variables.


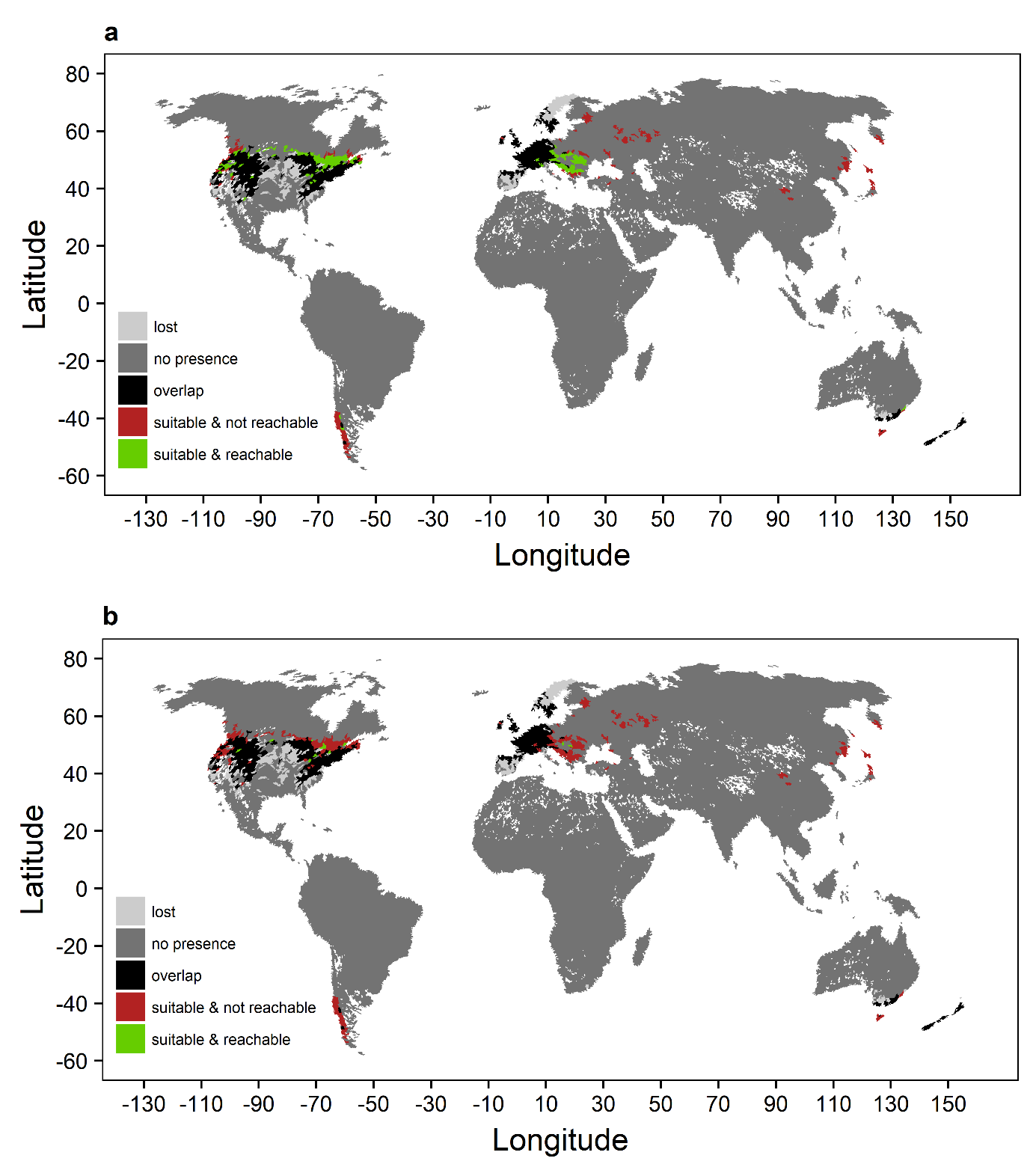


# Fig. S5 Distribution map of *Salmo trutta* (a) without and (b) with dams as dispersal barriers. Catchments with a presence in the baseline scenario that are suitable based on the predictions for the 2050s are marked as “overlap”, while catchments that are marked as “suitable & not reachable” cannot be reached either because of no available connection to a catchment with a current presence or due to dispersal barriers. Catchments that are predicted as not being suitable in the 2050s are marked as “lost”.


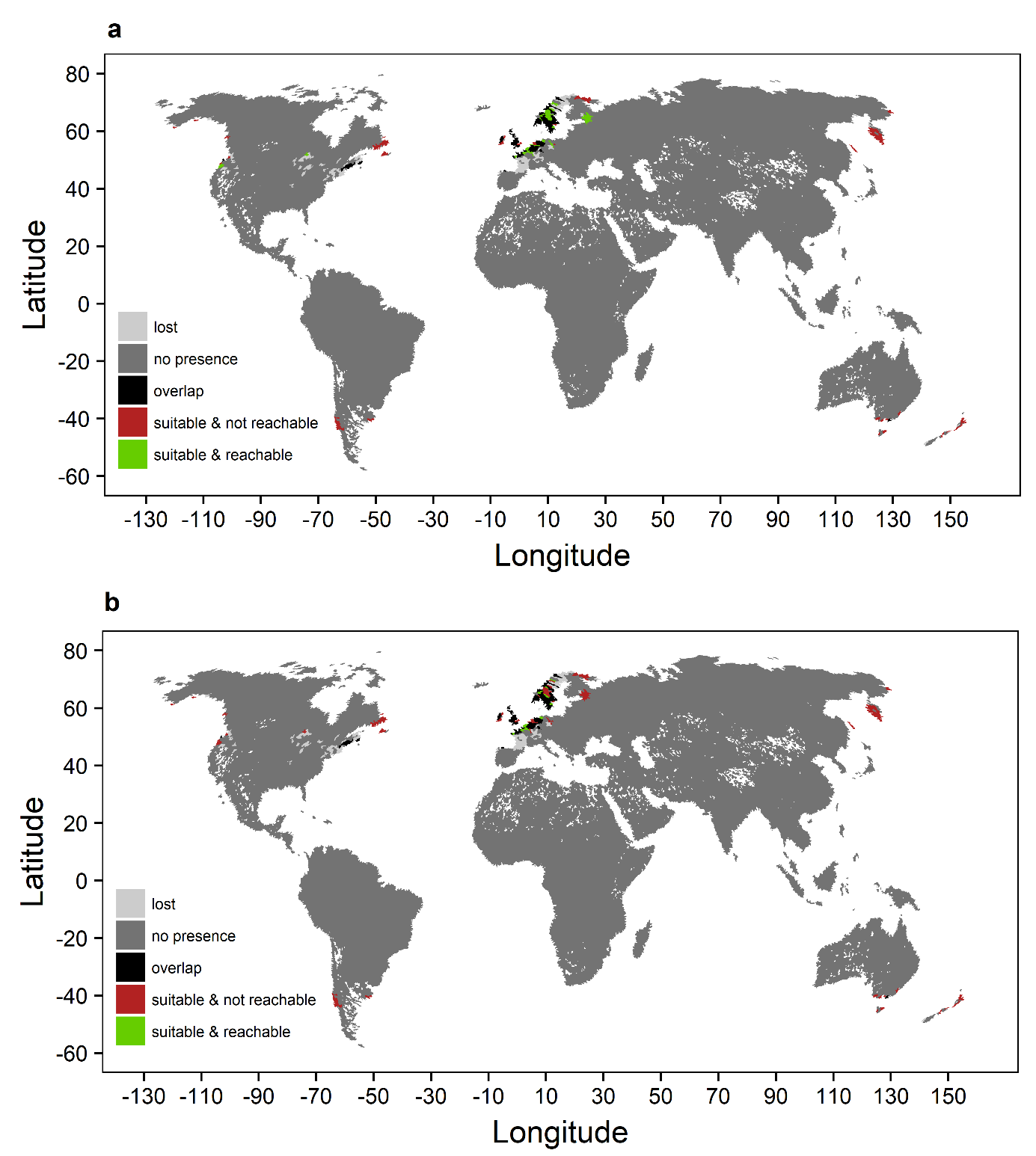


# Fig. S6 Distribution map of *Salmo salar* (a) without and (b) with dams as dispersal barriers. Catchments with a presence in the baseline scenario that are suitable based on the predictions for the 2050s are marked as “overlap”, while catchments that are marked as “suitable & not reachable” cannot be reached either because of no available connection to a catchment with a current presence or due to dispersal barriers. Catchments that are predicted as not being suitable in the 2050s are marked as “lost”.


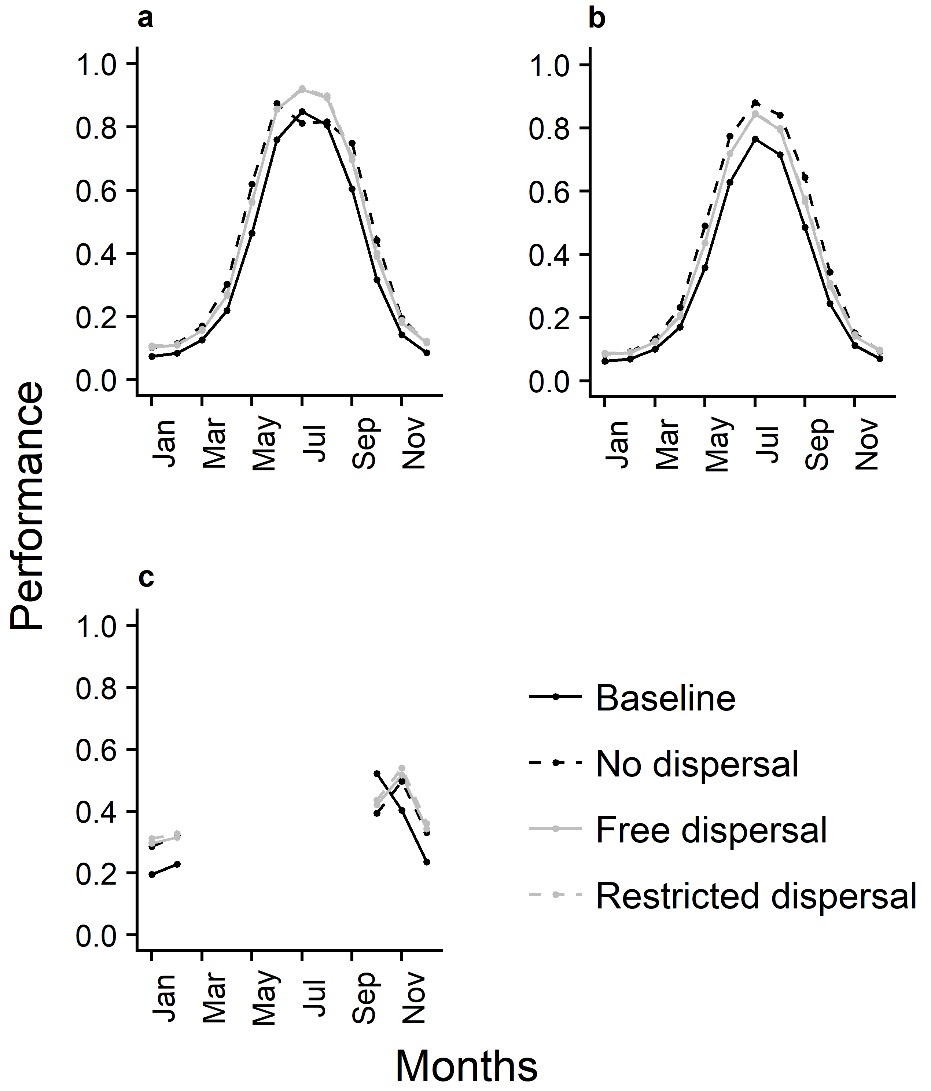


# Fig. S7 Baseline and 2050s monthly performance for the life stages (a) adults, (b) juveniles and (c) eggs of *Salmo salar* under consideration of different dispersal scenarios in the northern hemisphere. Analyses of the southern hemisphere were excluded because of few observations.

**
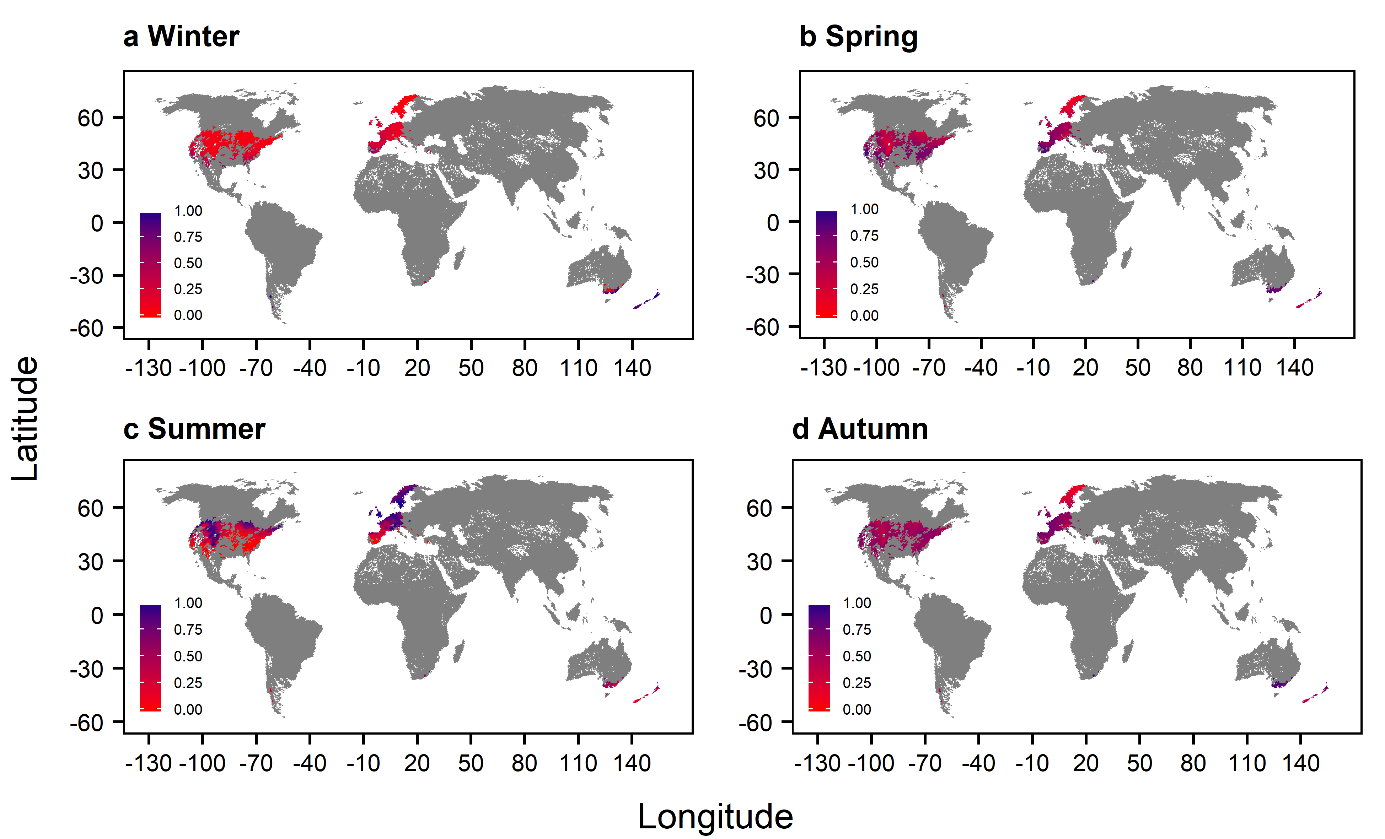
**

# Fig. S8 Global map of the seasonal performances of adult *Salmo trutta* for the “no dispersal” scenario. Note that seasons were defined according to the northern hemisphere.

**
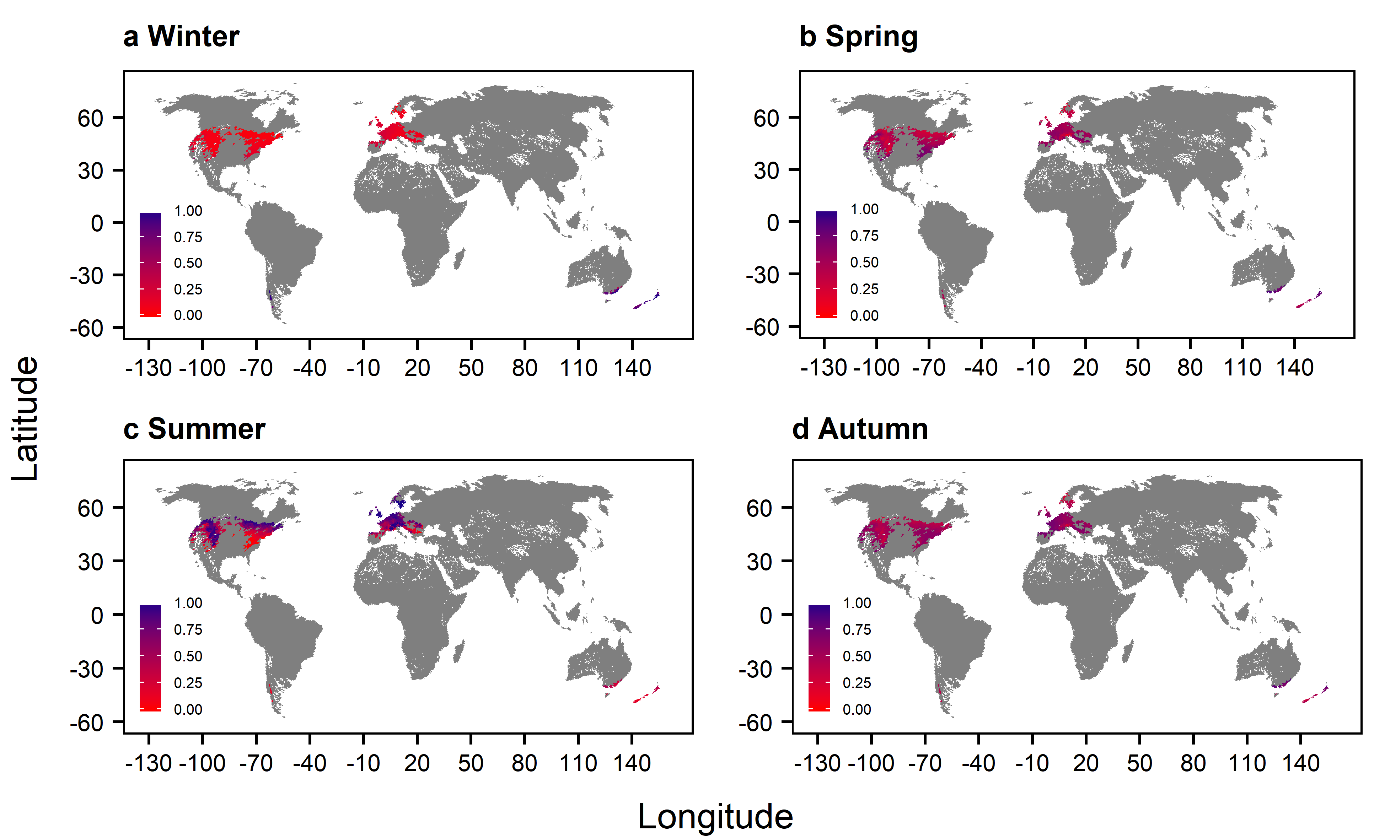
**

# Fig. S9 Global map of the seasonal performances of adult *Salmo trutta* for the “free dispersal” scenario. Note that seasons were defined according to the northern hemisphere.

**
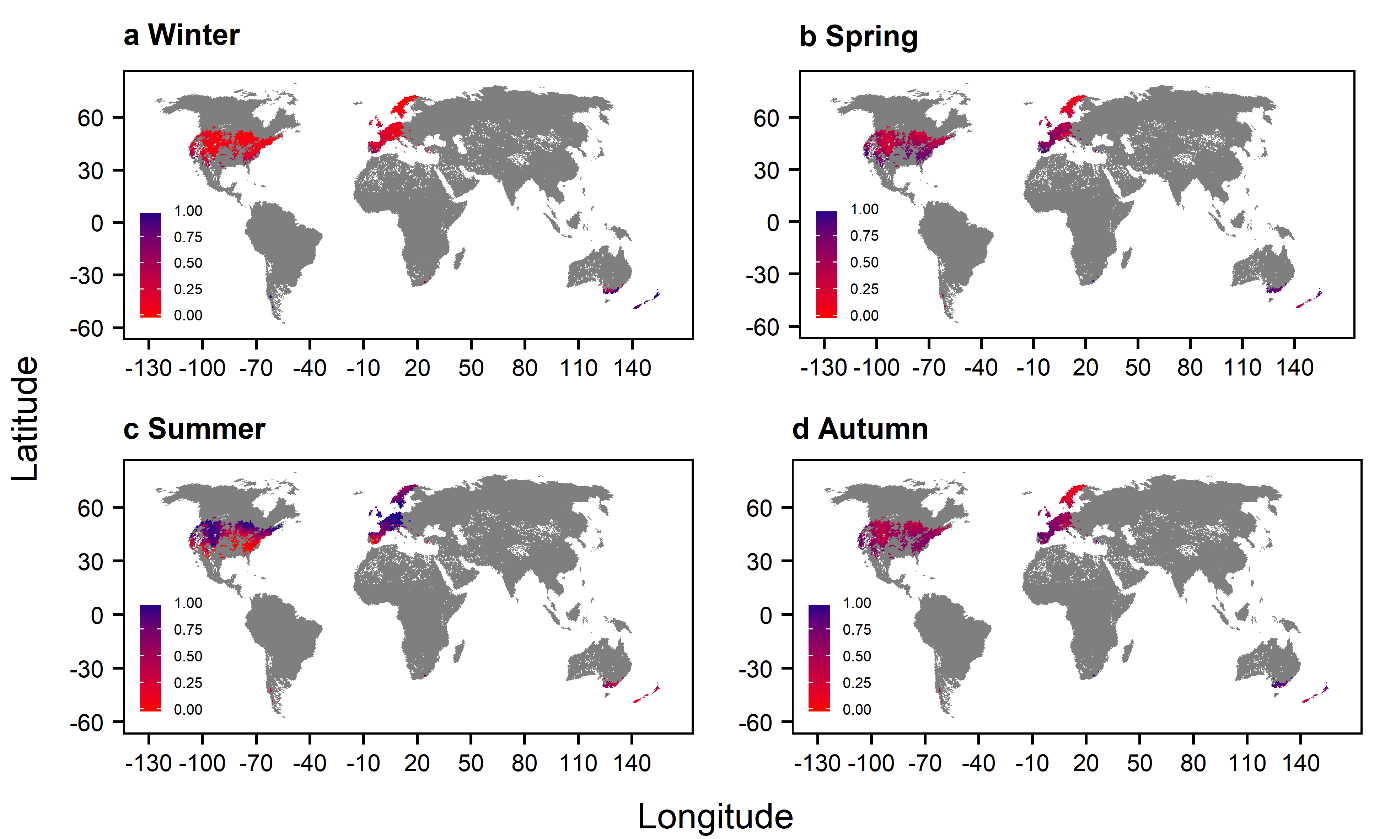
**

# Fig. S10 Global map of the seasonal performances of juvenile *Salmo trutta* for the “baseline” scenario. Note that seasons were defined according to the northern hemisphere.

**
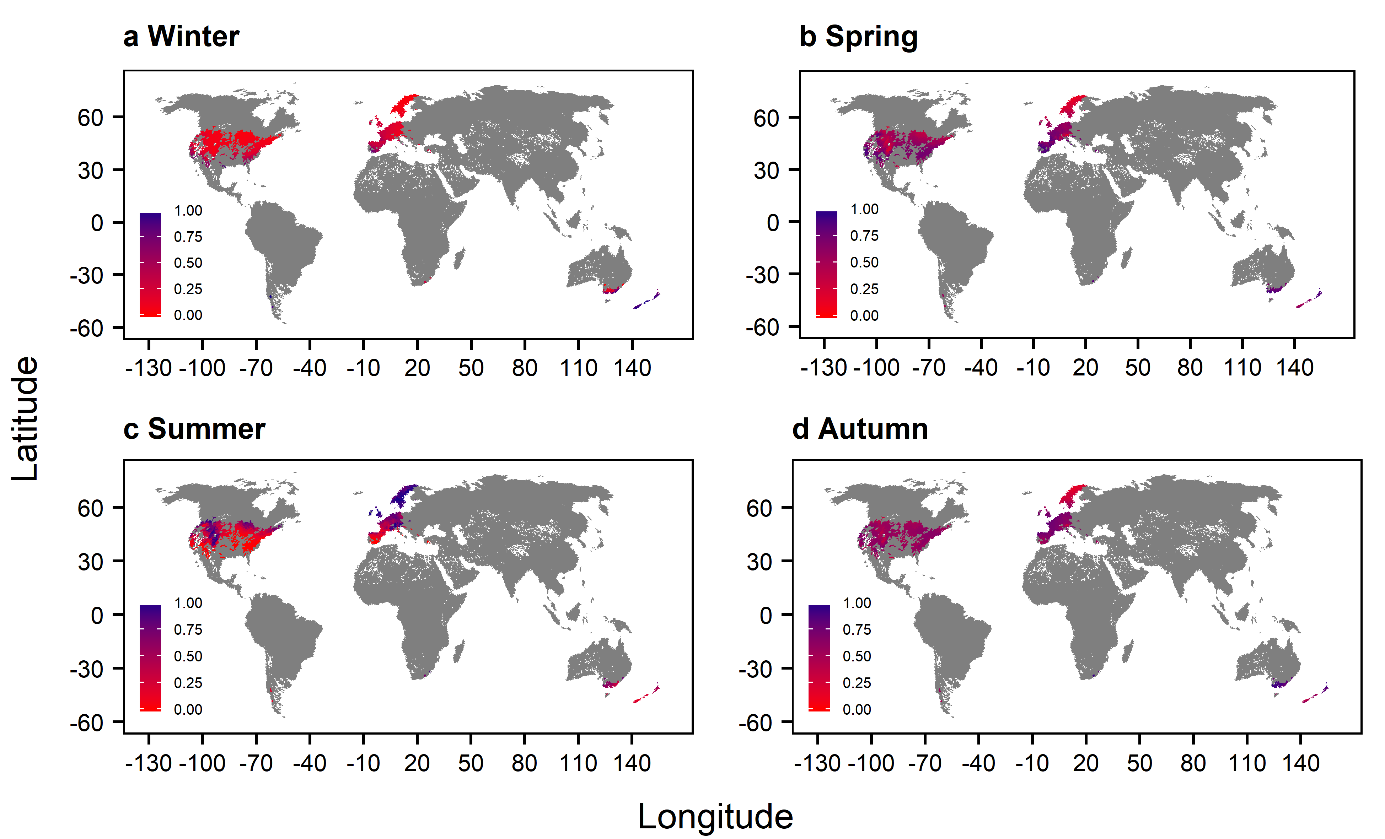
**

# Fig. S11 Global map of the seasonal performances of juvenile *Salmo trutta* for the “no dispersal” scenario. Note that seasons were defined according to the northern hemisphere.

**
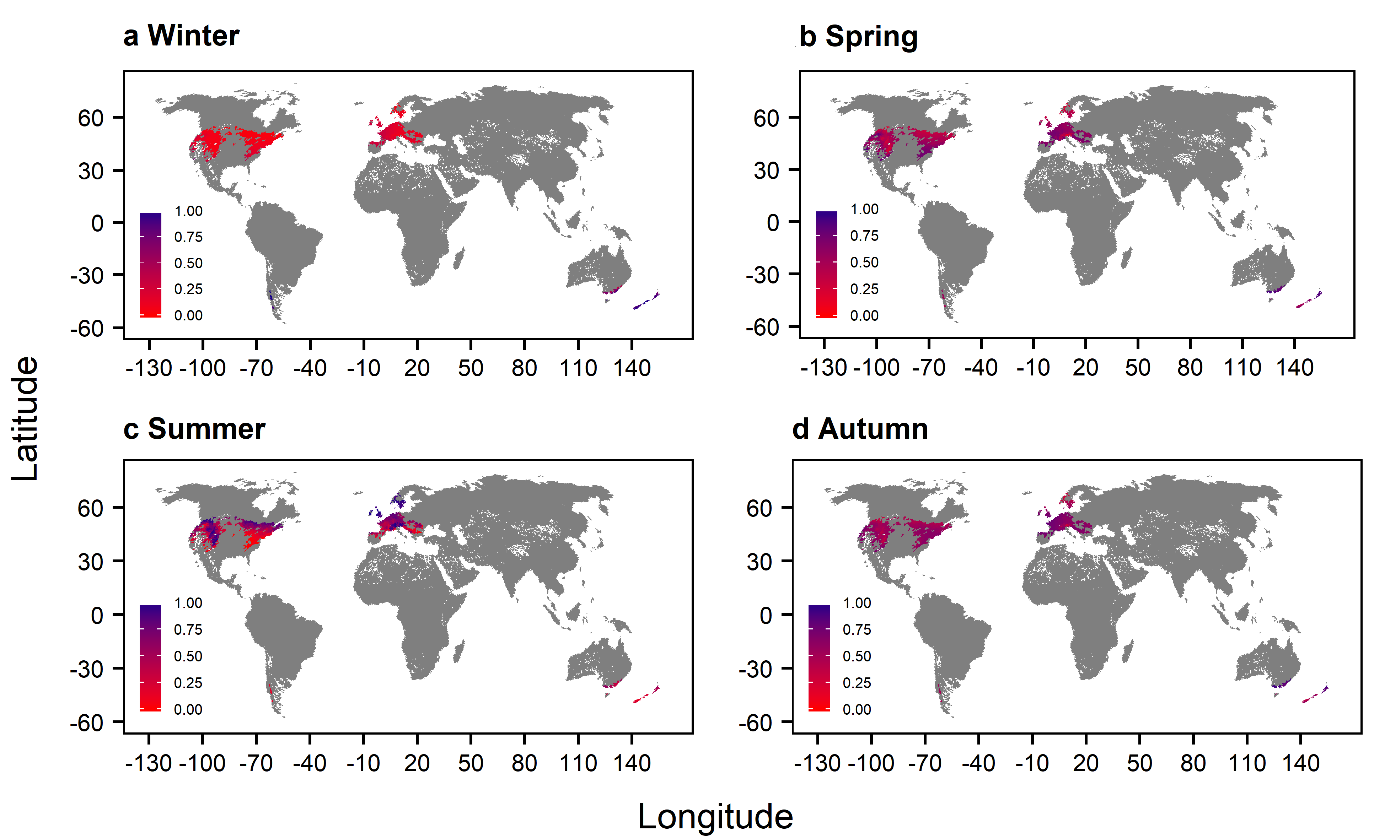
**

# Fig. S12 Global map of the seasonal performances of juvenile *Salmo trutta* for the “free dispersal” scenario. Note that seasons were defined according to the northern hemisphere.

**
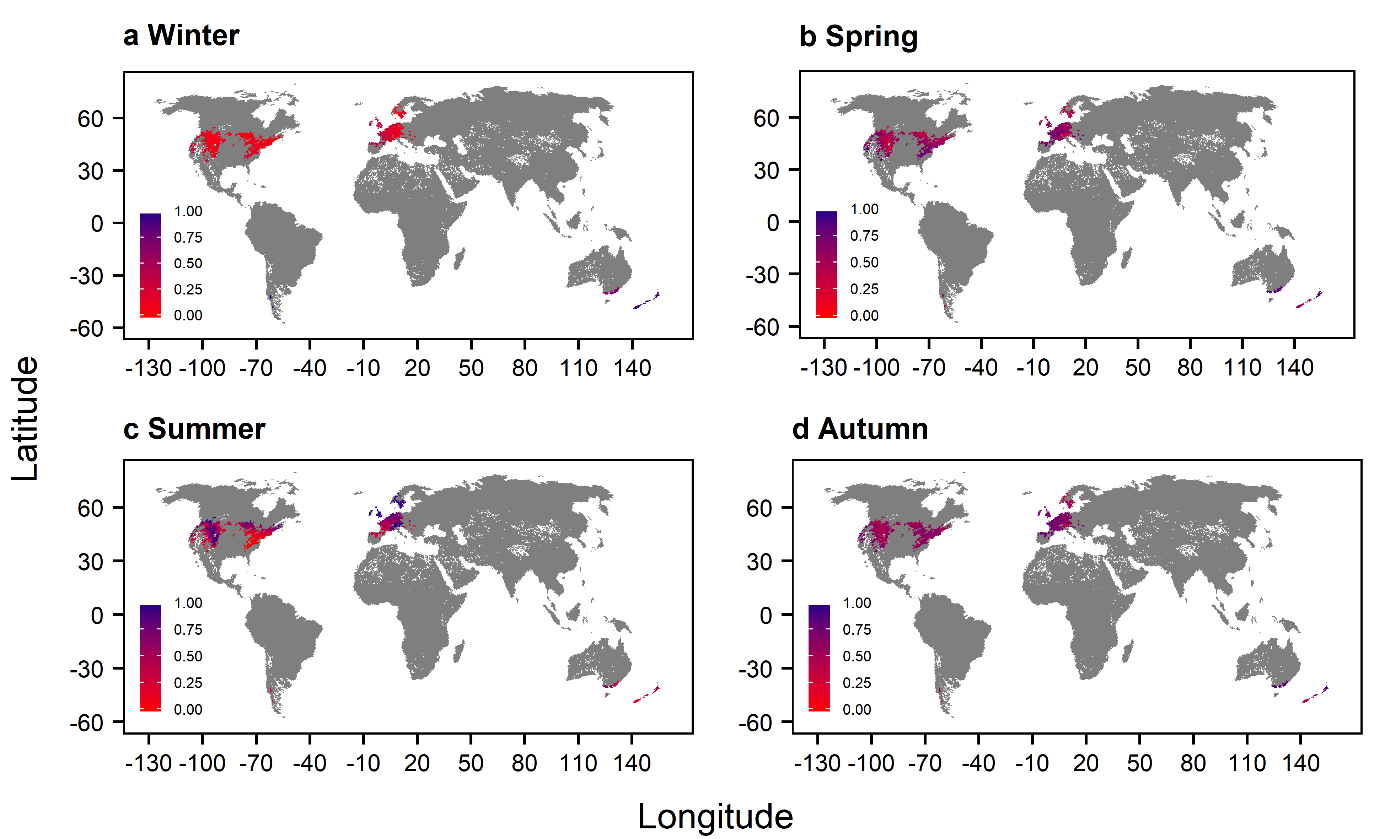
**

# Fig. S13 Global map of the seasonal performances of juvenile *Salmo trutta* for the “restricted dispersal” scenario. Note that seasons were defined according to the northern hemisphere.

**
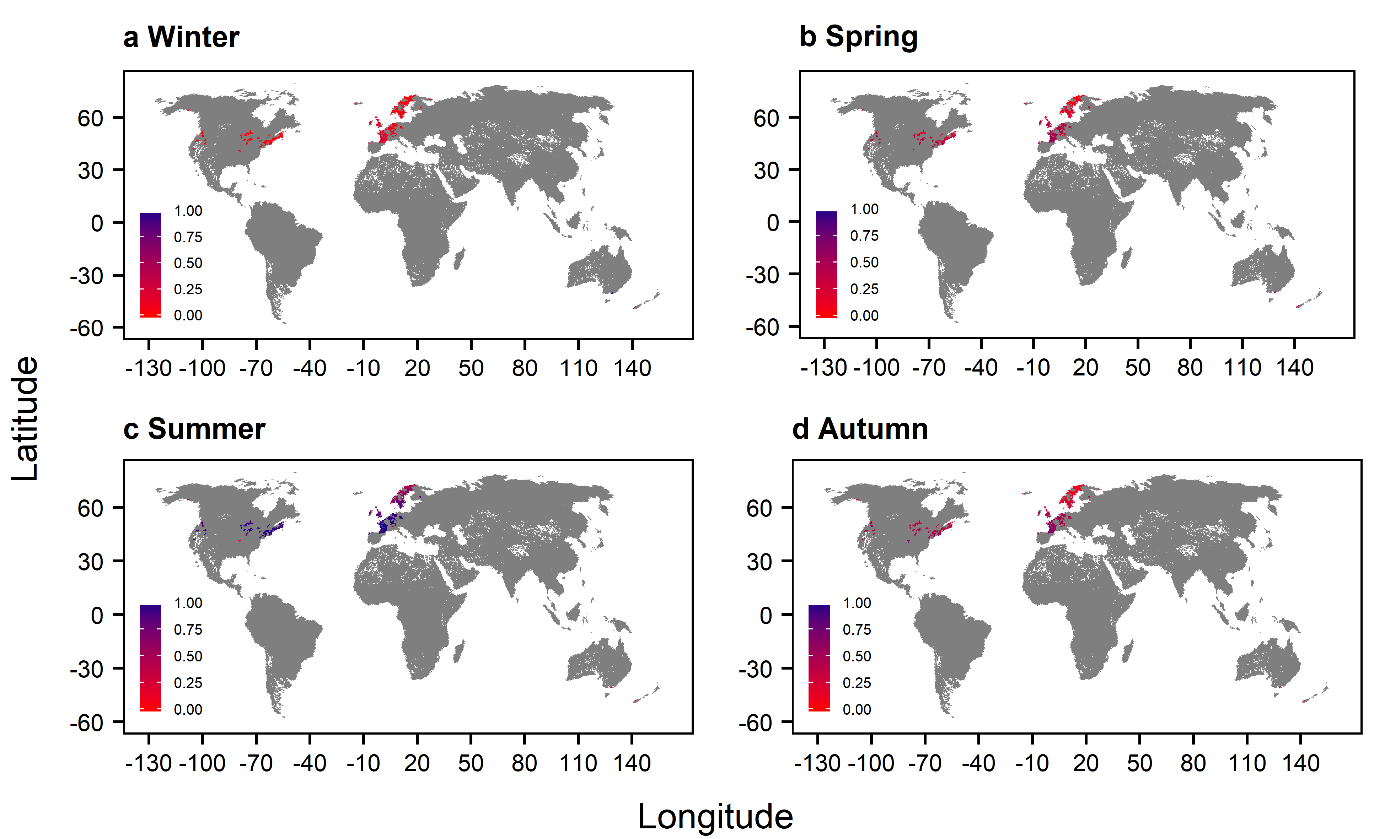
**

# Fig. S14 Global map of the seasonal performances of adult *Salmo salar* for the “baseline” scenario. Note that seasons were defined according to the northern hemisphere.

**
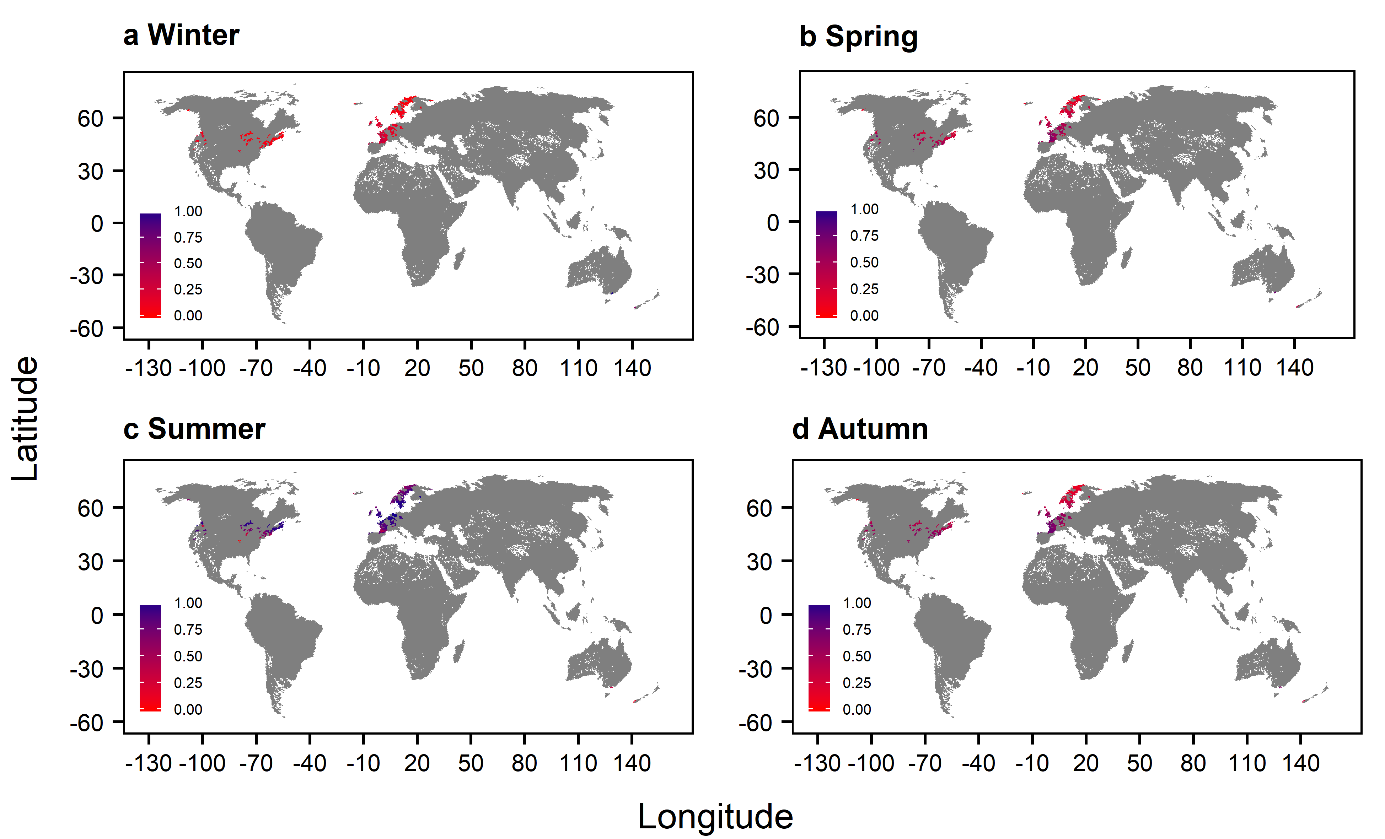
**

# Fig. S15 Global map of the seasonal performances of adult *Salmo salar* for the “no dispersal” scenario. Note that seasons were defined according to the northern hemisphere.

**
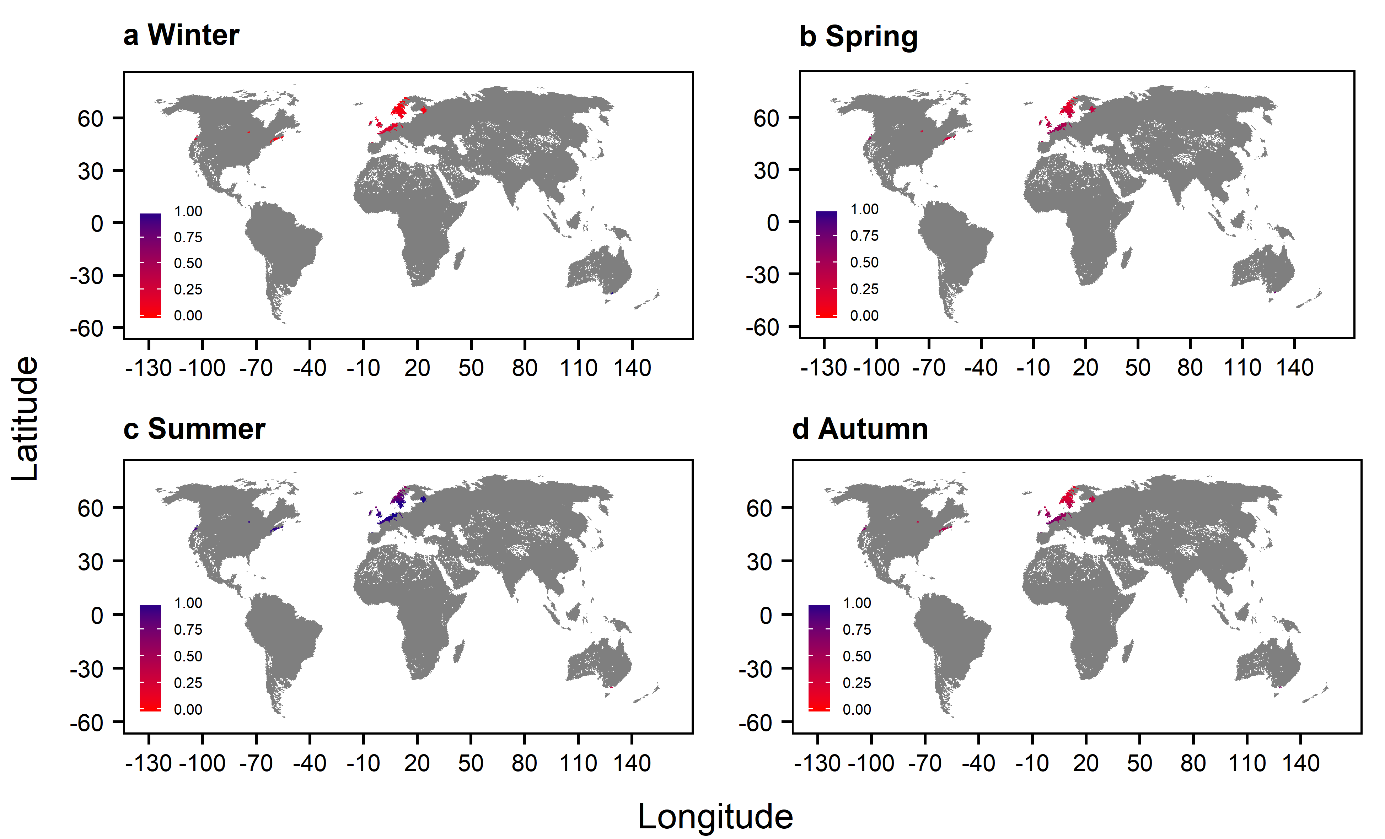
**

# Fig. S16 Global map of the seasonal performances of adult *Salmo salar* for the “free dispersal” scenario. Note that seasons were defined according to the northern hemisphere.

**
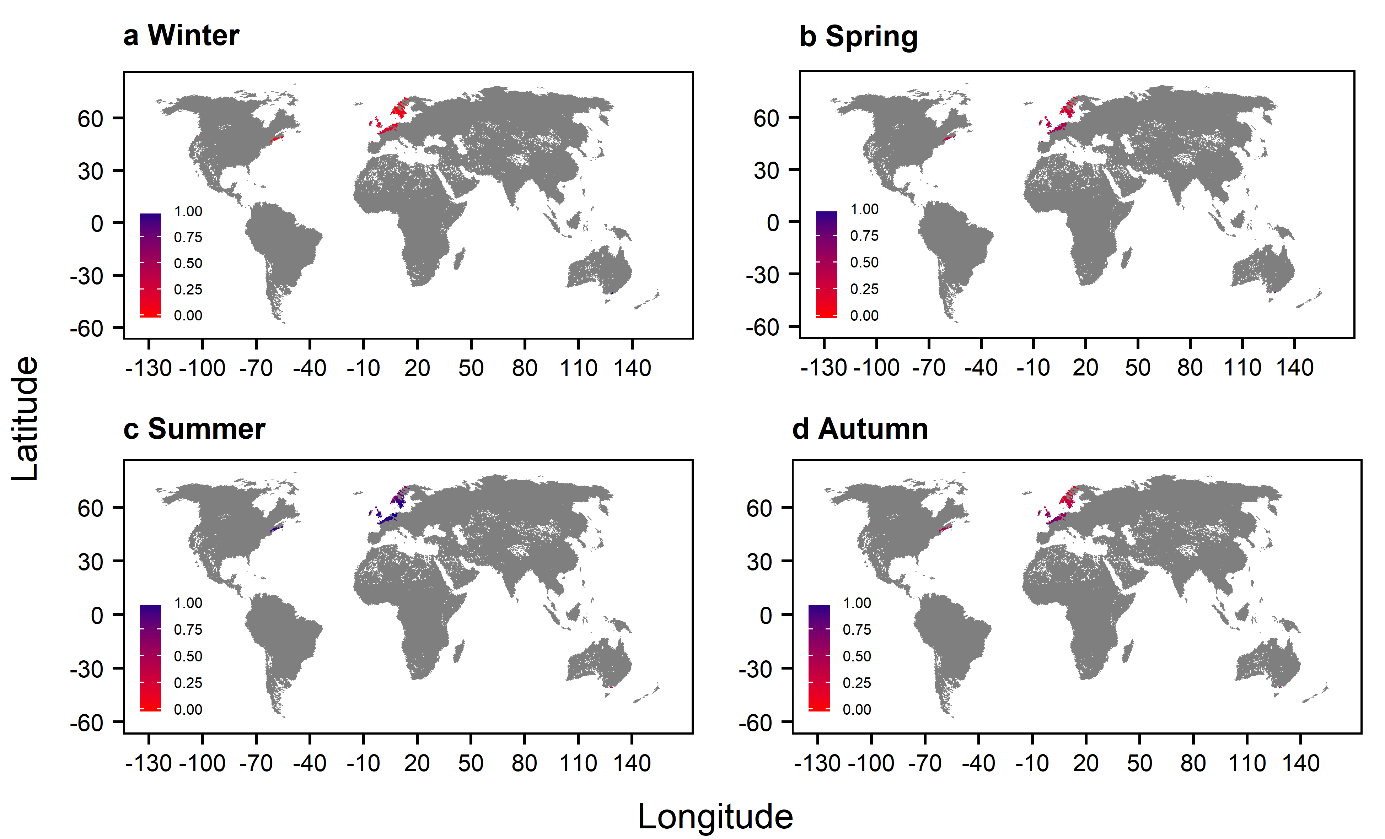
**

# Fig. S17 Global map of the seasonal performances of adult *Salmo salar* for the “restricted dispersal” scenario. Note that seasons were defined according to the northern hemisphere.

**
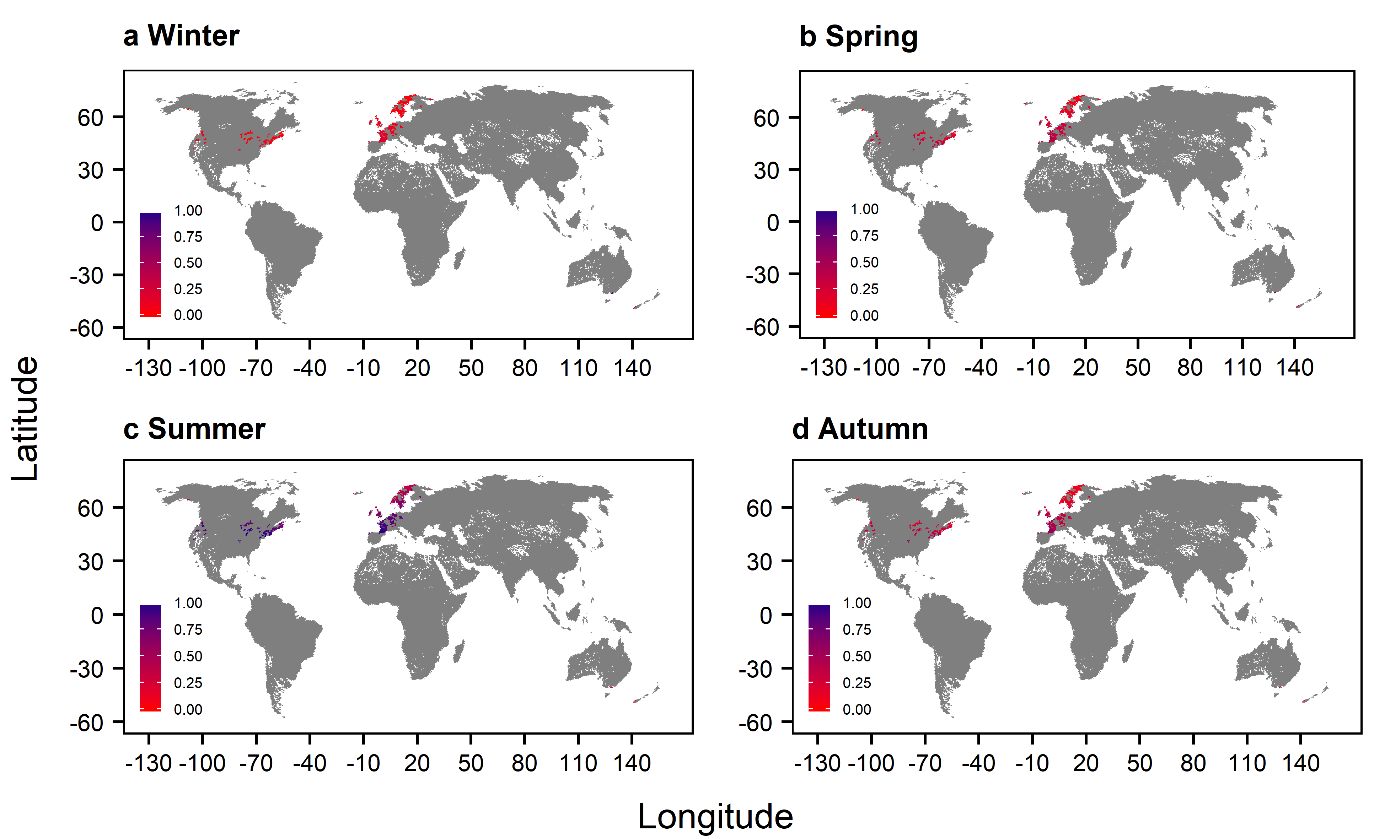
**

# Fig. S18 Global map of the seasonal performances of juvenile *Salmo salar* for the “baseline” scenario. Note that seasons were defined according to the northern hemisphere.

**
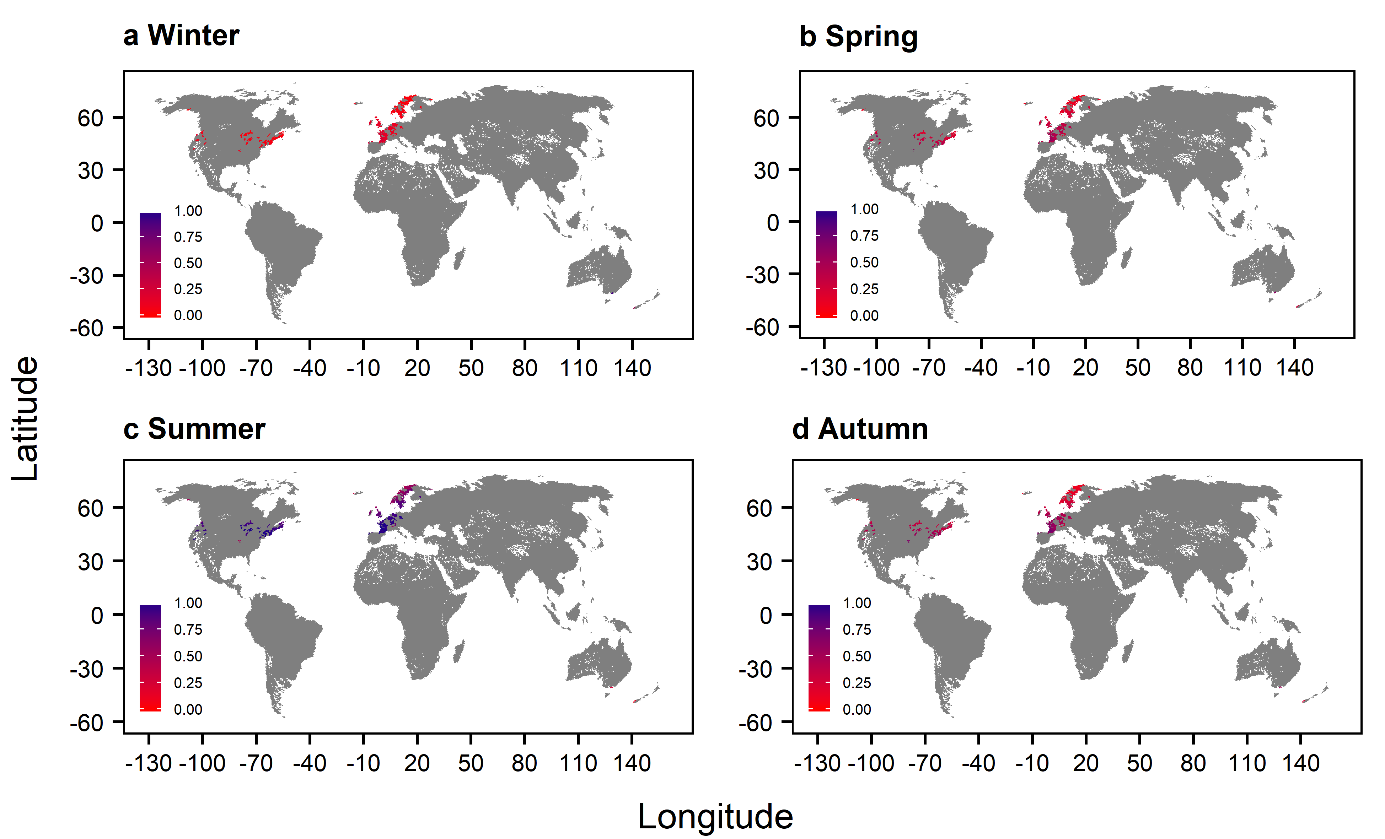
**

# Fig. S19 Global map of the seasonal performances of juvenile *Salmo salar* for the “no dispersal” scenario. Note that seasons were defined according to the northern hemisphere.

**
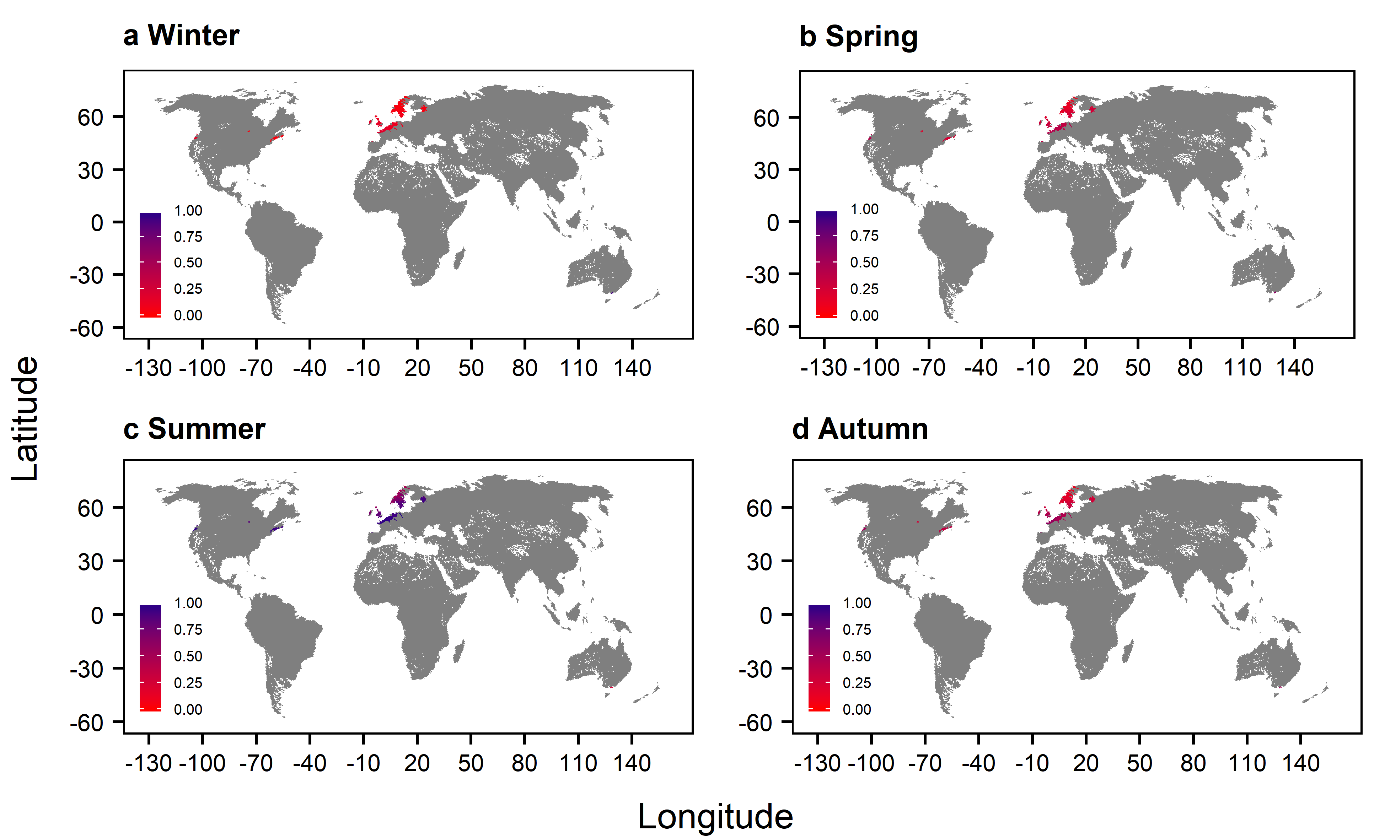
**

# Fig. S20 Global map of the seasonal performances of juvenile *Salmo salar* for the “free dispersal” scenario. Note that seasons were defined according to the northern hemisphere.

**
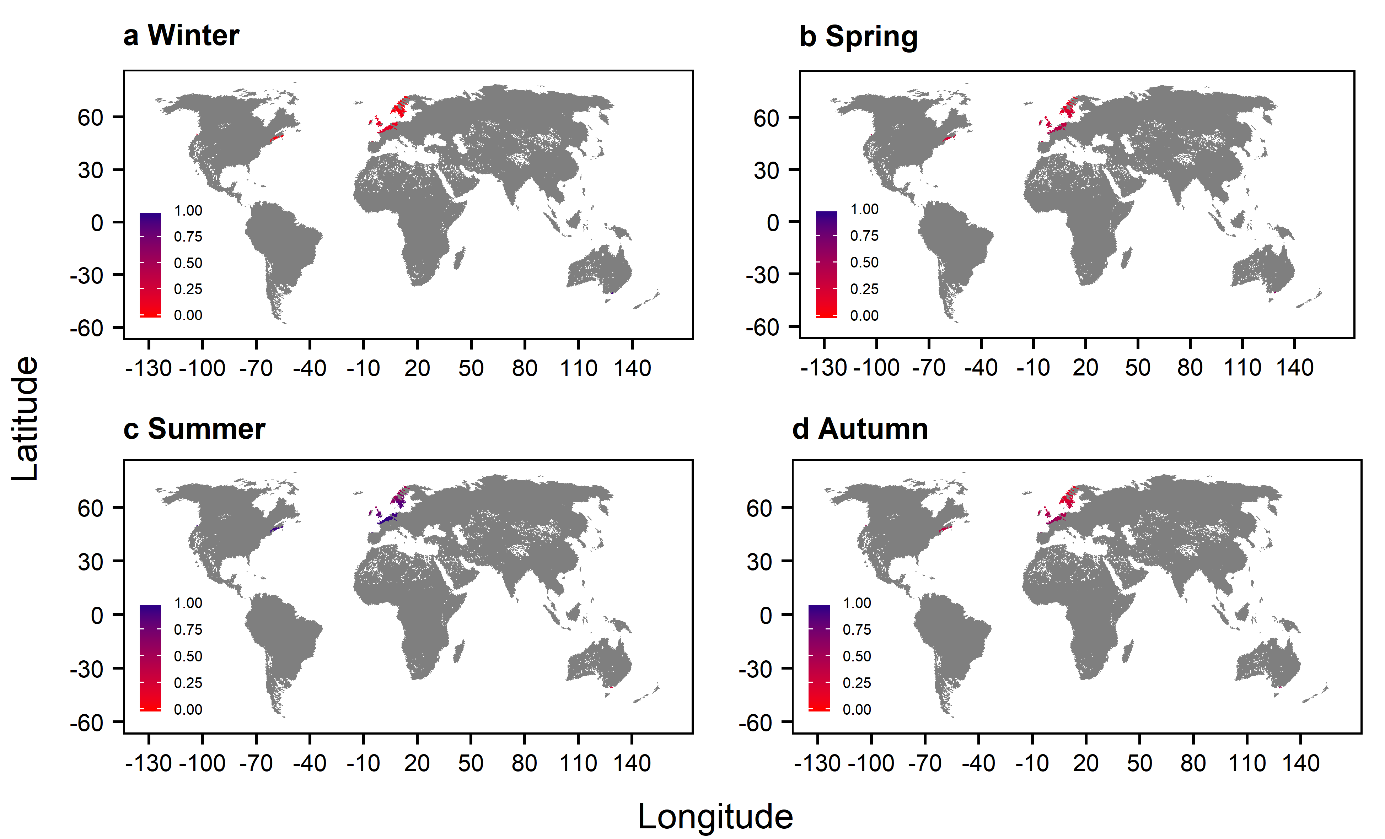
**

# Fig. S21 Global map of the seasonal performances of juvenile *Salmo salar* for the “restricted dispersal” scenario. Note that seasons were defined according to the northern hemisphere.


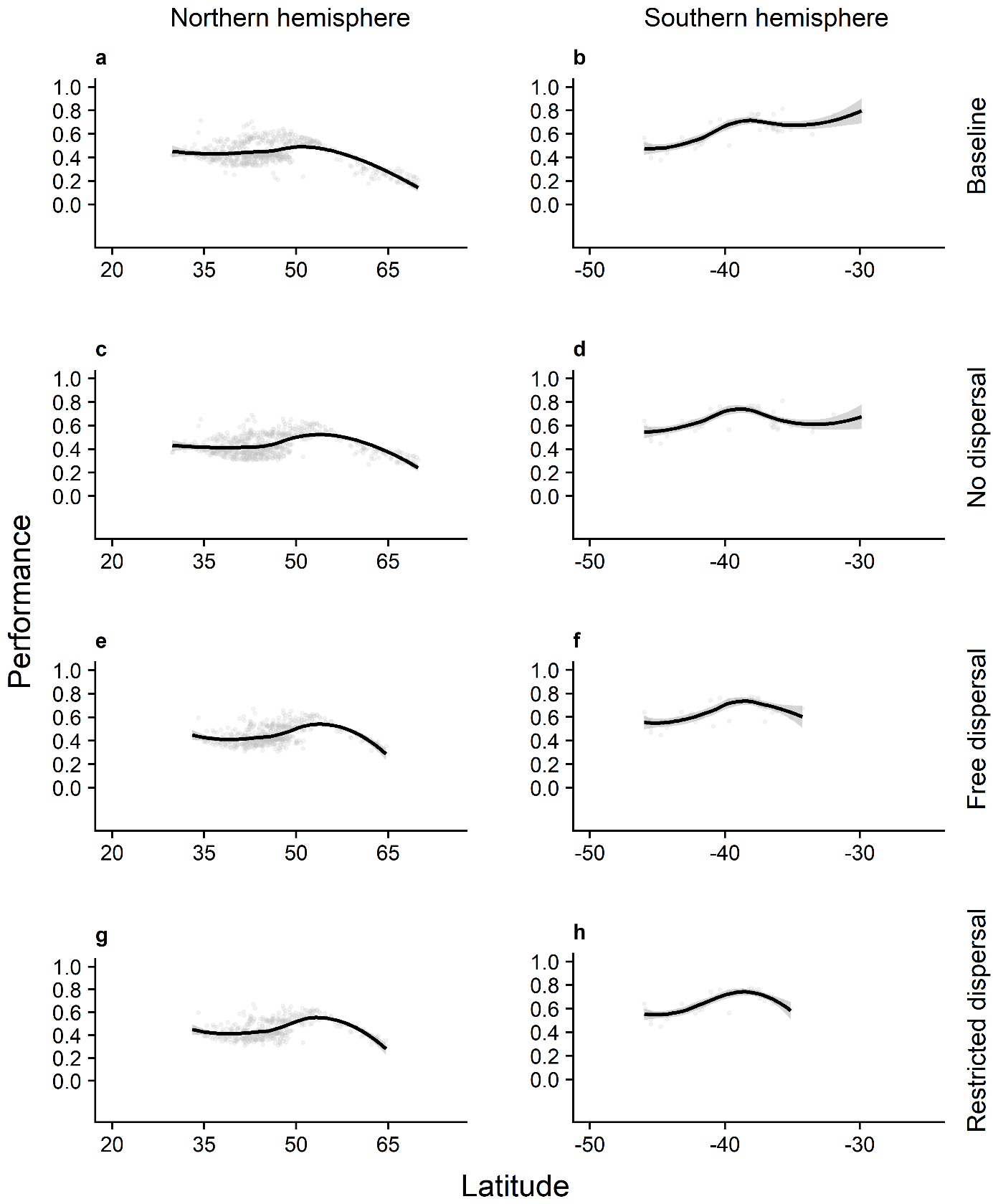


# Fig. S22 Latitudinal trends of annual mean performance for juvenile *Salmo trutta* under consideration of different dispersal scenarios. Annual mean performance is based on monthly performance values.


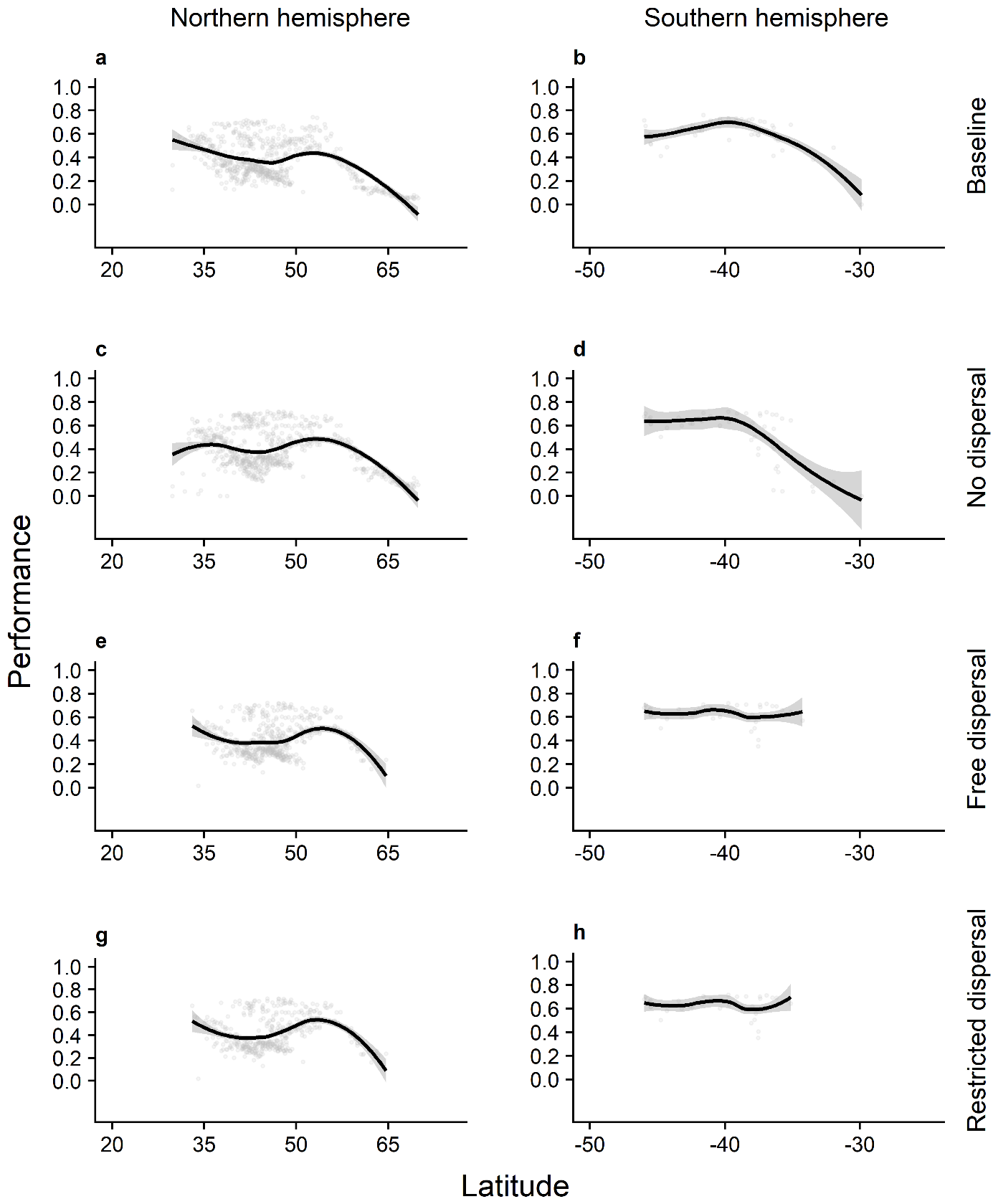


# Fig. S23 Latitudinal trends of the performance during the spawning seasons for *Salmo trutta* eggs under consideration of different dispersal scenarios. Performance is based on monthly performance values.

**
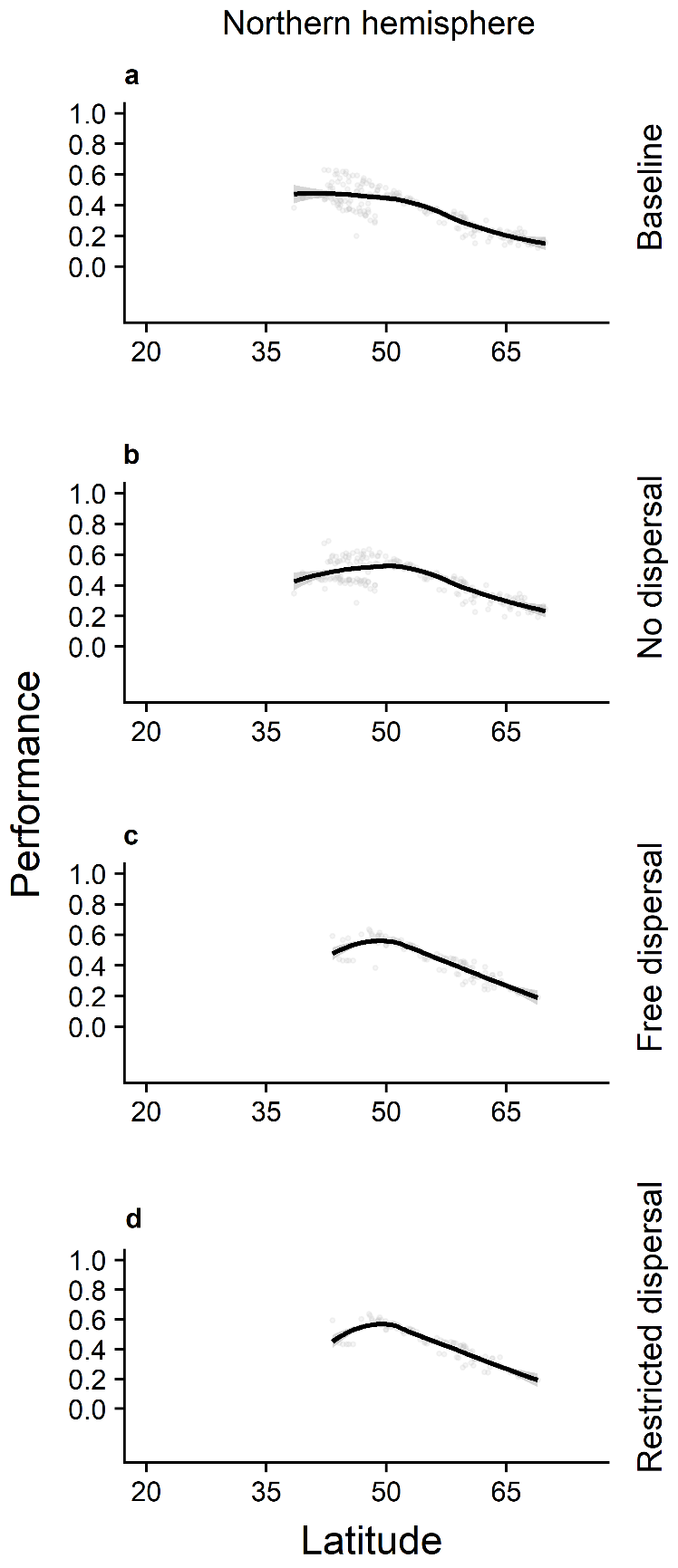
**

# Fig. S24 Latitudinal trends of annual mean performance for adult *Salmo salar* under consideration of different dispersal scenarios in the norther hemisphere. Annual mean performance is based on monthly performance values. Analyses of the southern hemisphere were excluded because of few observations.


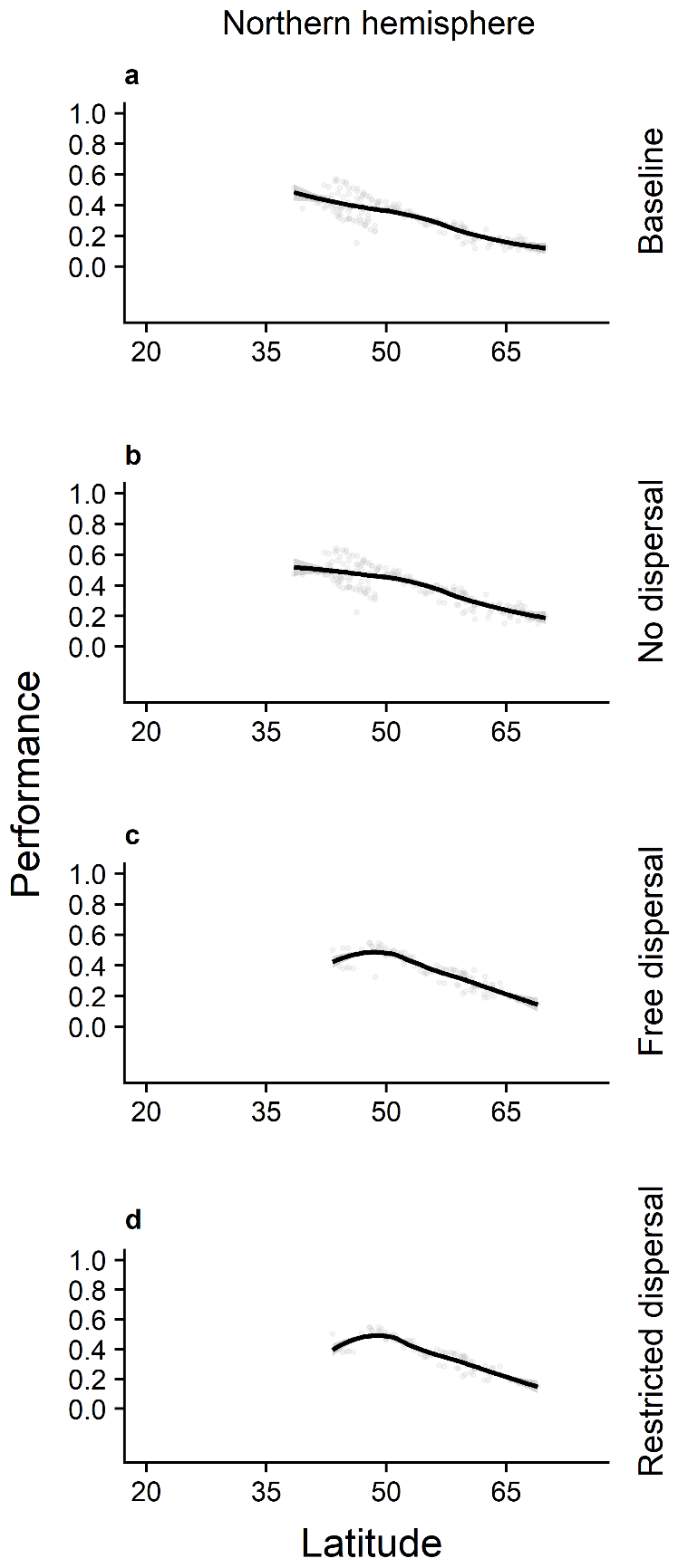


# Fig. S25 Latitudinal trends of annual mean performance for juvenile *Salmo salar* under consideration of different dispersal scenarios in the norther hemisphere. Annual mean performance is based on monthly performance values. Analyses of the southern hemisphere were excluded because of few observations.


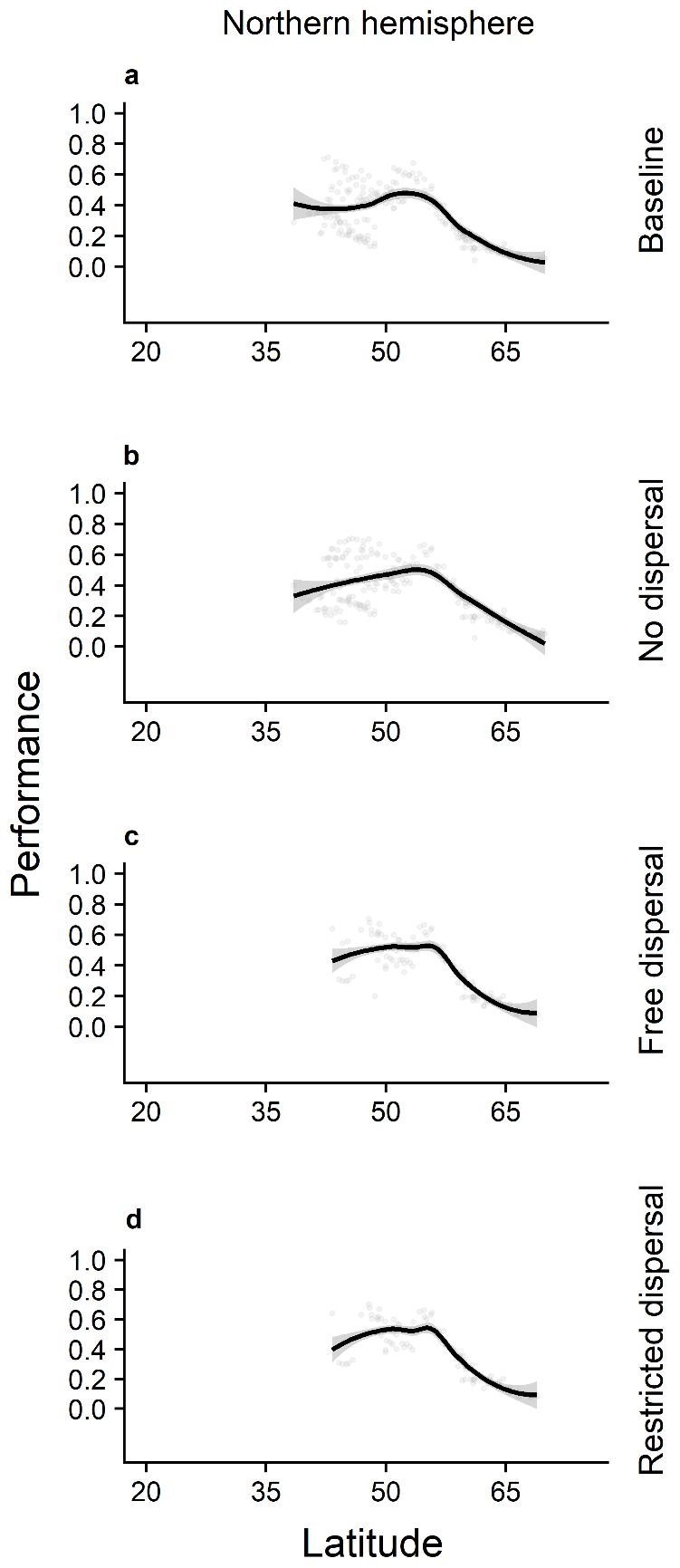


# Fig. S26 Latitudinal trends of the performance during the spawning season for *Salmo salar* eggs under consideration of different dispersal scenarios in the norther hemisphere. Performance is based on monthly performance values. Analyses of the southern hemisphere were excluded because of few observations.

# Table S1 Overview of the catchment and species occurrence numbers per region.

| **Region** | **Total Catchment Number** | **Species occurrence number** | |
| --- | --- | --- | --- |
|  |  | ***Salmo trutta*** | ***Salmo salar*** |
| Africa | 2,687 | 2 | 0 |
| Asia | 3,981 | 0 | 0 |
| Australia | 725 | 49 | 2 |
| Europe | 921 | 293 | 147 |
| North America | 1,829 | 384 | 50 |
| South America | 1,552 | 2 | 0 |
| **Sum** | 11,695 | 730 | 199 |

# Table S2 Overview of the initially considered environmental variables.

| **Category** | **Variable (abbr.)** | **Description** | **Literature** |
| --- | --- | --- | --- |
| Climatic  (discharge) | Annual mean discharge (AnnMeanDis) | Average of the annual mean discharges | e.g. Laizé et al., 2014; Markovic et al., 2017 |
|  | Maximum discharge (MaxDis) | Average of the annual maximum discharges |  |
|  | Minimum discharge (MinDis) | Average of the annual minimum discharges |  |
|  | Discharge seasonality (DisSeas) | Average of the annual standard deviation of discharges |  |
|  | Flow of the wettest month (FloWetMonth) | Maximum of the monthly discharges |  |
|  | Flow of the driest month (FloDriMonth) | Minimum of the monthly discharges |  |
|  | Mean winter discharge (WinDis) | Mean discharge for the months December – February | e.g. Wenger et al., 2011a; Wenger et al., 2011b |
|  | Mean spring discharge (SpriDis) | Mean discharge for the months March – May |  |
|  | Mean summer discharge (SumDis) | Mean discharge for the months June – August |  |
|  | Mean autumn discharge (AutDis) | Mean discharge for the months September – November |  |
| Climatic  (water temperature) | Annual mean water temperature (AnnMeanWaT) | Average of the annual mean water temperatures | e.g. Markovic et al. 2012; Markovic et al. 2014 |
|  | Maximum water temperature (MaxWaT) | Average of the annual maximum water temperatures |  |
|  | Minimum water temperature (MinWaT) | Average of the annual minimum water temperatures |  |
|  | Water temperature seasonality (WaTSeas) | Average of the annual standard deviation of water temperatures |  |
|  | Water temperature of the warmest month (WaTWarmMonth) | Maximum of the monthly water temperatures |  |
|  | Water temperature of the coldest month (WaTColdMonth) | Minimum of the monthly water temperatures |  |
|  | Mean winter water temperature (WinWaT) | Mean water temperature for the months December – February |  |
|  | Mean spring water temperature (SpriWaT) | Mean water temperature for the months March – May |  |
|  | Mean summer water temperature (SumWaT) | Mean water temperature for the months June – August |  |
|  | Mean autumn water temperature (AutWaT) | Mean water temperature for the months September – November |  |
|  | Mean diurnal range (MeanDiuRan) | Mean of monthly (maximum – minimum water temperature) |  |
|  | Annual water temperature range (AnnWaTRan) | Maximum water temperature – minimum water temperature |  |
|  | Isothermality (Isotherm) | Mean diurnal range / Annual water temperature range |  |
| Topographic | Altitude (Alt) | Mean catchment elevation | e.g. McNyset, 2005 |
| Land cover | Cropland (Crop) | Percentage of catchment area covered by cropland | e.g. Trautwein et al., 2012 |
|  | Built-up area (Built-up) | Fraction of sealed areas within the catchment |  |
|  | Forest (Forest) | Percentage of catchment area covered by forest |  |
|  | Grassland (Gras) | Percentage of catchment area covered by grassland |  |
|  | Shrubland (Shrub) | Percentage of catchment area covered by shrubland |  |

# Table S3 Thermal traits of *Salmo trutta* for different life stages.

| **Life stage** | **CT_min_** | **CT_max_** | **T_opt_** | **Literature** |
| --- | --- | --- | --- | --- |
| adults | 0 | 30.0 | 17 | Küttel et al., 2002 |
|  |  | 26.0 | 19 | Otto & Zahn, 2008 |
|  |  | 29.0 |  | Lee & Rinne, 1980 |
|  |  | 29.9 |  | Lee & Rinne, 1980 |
|  | 0 | 24.7 |  | Jonsson & Jonsson, 2009 |
|  |  | 23.0 | 19 | Otto & Zahn, 2008 |
|  |  | 25.0 |  | Otto & Zahn, 2008 |
|  | 0 | 30.0 | 17 | Küttel et al., 2002 |
|  |  | 29.0 |  | Beitinger et al., 2000 |
|  |  | 29.8 |  | Beitinger et al., 2000 |
|  |  | 30.0 |  | Beitinger et al., 2000 |
|  |  | 24.6 |  | Todd et al., 2008 |
|  |  | 29 |  | Lee & Rinne, 1980 |
|  |  | 29.9 |  | Lee & Rinne, 1980 |
| juveniles |  | 26.3 |  | Grande & Andersen, 1991 |
|  |  | 27.8 |  | Grande & Andersen, 1991 |
|  | 0 | 28.0 | 14 | Küttel et al., 2002 |
|  |  | 23.2 |  | Yoder et al., 2012 |
|  |  | 26.0 |  | Yoder et al., 2012 |
|  |  | 26.4 |  | Yoder et al., 2012 |
|  |  |  | 17 | Jonsson & Jonsson, 2009 |
|  | 0 | 28.0 | 14 | Küttel et al., 2002 |
|  |  | 27.8 |  | Grande & Andersen, 1991 |
| eggs | 0 | 15.0 | 7 | Küttel et al., 2002 |
|  | 0 | 12.0 | 9 | Küttel et al., 2002 |
|  |  | 16.0 |  | Ojanguren & Braña, 2003 |
|  |  | 18.0 |  | Ojanguren & Braña, 2003 |
|  |  |  | 8 | Ojanguren & Braña, 2003 |
|  |  |  | 10 | Ojanguren & Braña, 2003 |
|  | 0 | 15.0 | 7 | Küttel et al., 2002 |

# Table S4 Thermal traits of *Salmo salar* for different life stages.

| **Life stage** | **CT_min_** | **CT_max_** | **T_opt_** | **Literature** |
| --- | --- | --- | --- | --- |
| adults |  | 29.0 | 17 | Küttel et al., 2002 |
|  |  |  | 20 | Küttel et al., 2002 |
|  |  |  |  | Küttel et al., 2002 |
|  | -0.5 | 25.0 | 17 | Wallace, 1993 |
|  |  | 29.0 | 15 | Otto & Zahn, 2008 |
|  | 0 | 27.8 |  | Jonsson & Jonsson, 2009 |
|  |  | 28.0 | 15 | Otto & Zahn, 2008 |
|  |  | 34.0 | 17 | Küttel et al., 2002 |
| juveniles | 0 | 28.0 | 23 | Küttel et al., 2002 |
|  |  | 32.9 |  | Beitinger et al., 2000 |
|  |  | 32.8 |  | Beitinger et al., 2000 |
|  |  | 32.6 |  | Beitinger et al., 2000 |
|  |  | 32.7 |  | Beitinger et al., 2000 |
|  |  |  | 20 | Jonsson & Jonsson, 2009 |
|  |  | 28.7 |  | Grande & Andersen, 1991 |
|  |  | 29.2 |  | Grande & Andersen, 1991 |
|  |  | 30.0 |  | Garside, 1973 |
|  |  | 28.5 |  | Garside, 1973 |
|  |  | 29.0 |  | Garside, 1973 |
| eggs | 0 | 16.0 | 11 | Küttel et al., 2002 |
|  | 0 | 12.0 |  | Otto & Zahn, 2008 |
|  | 0 | 16.0 |  | Elliott, 1981 |

# Table S5 Parameter tuning specifications for Artificial Neural Networks (ANN, manual and ADADELTA), Random Forest (RF), Gradient Boosting Machines (GBM), Multivariate Adaptive Regression Splines (MARS) and Elastic Net (ELNET).

| **Method** | **Parameter** | **Min** | **Max** | **Steps** | **Explanation** |
| --- | --- | --- | --- | --- | --- |
| ANN  (manual) | Activation function |  |  |  | Activation functions define the output of a neuron. Three different functions were tested: Rectifier, Tanh, Maxout. |
|  | Number of nodes | 5 | 500 | "+" 5 | Number of nodes within the single hidden layer. |
|  | Learning rate | 0.001 | 0.1 | "+" 0.001 | The learning rate defines the step size during gradient descent. |
|  | Learning rate annealing | 1.00E-08 | 1.00E-05 | "×" 10 | The annealing of the learning rate avoids the risk of jumping around local minima. |
|  | Learning rate decay factor | 0.8 | 1 | "+" 0.05 | Decay factor of the learning rate between layers. |
|  | Initial momentum | 0 | 0.8 | "+" 0.1 | Accelerates the gradient descent by using gradient information from previous iterations. |
|  | Final momentum | 0.9 | 0.99 | "+" 0.01 | Final momentum after a certain number of training samples for which the momentum increases. |
|  | Dropout ratio | 0 | 0.2 | "+" 0.05 | Input layer dropout ratio. |
|  | L1-regularization | 0 | 1.00E-04 | "+" 1.0E-06 | Lambda value for Lasso regularization. |
|  | L2-regularization | 0 | 1.00E-04 | "+" 1.0E-06 | Lambda value for Ridge regularization. |
|  | Epochs | 10 | 1.00E+03 |  | Number of iterations of the whole dataset. The following values were used: 10, 250, 500, 750, 1000. |
|  | MaxW² | 10 | 3.40E+38 |  | Constraint for squared sum of incoming weights per unit. Following values were used: 10, 100, 1000, 3.4028235e+38 (default). |
|  | Balanced classes |  |  |  | Balanced classes set to TRUE in order to balance the class distribution by either oversample or undersample the minority class. |
| ANN  (ADADELTA) | Activation function |  |  |  | Activation functions define the output of a neuron. Three different functions were tested: Rectifier, Tanh, Maxout. |
|  | Number of nodes | 5 | 500 | "+" 5 | Number of nodes within the single hidden layer. |
|  | Rho | 0.9 | 0.999 | "+" 0.001 | Rho is similar to prior weight updates (similar to momentum). |
|  | Epsilon | 1.00E-09 | 1.00E-03 | "×" 10 | Epsilon is similar to learning rate annealing during initial training and at later stages to momentum. |
|  | Dropout ratio | 0 | 0.2 | "+" 0.05 | Input layer dropout ratio. |
|  | L1-regularization | 0 | 1.00E-04 | "+" 1.0E-06 | Lambda value for Lasso regularization. |
|  | L2-regularization | 0 | 1.00E-04 | "+" 1.0E-06 | Lambda value for Ridge regularization. |
|  | Epochs | 10 | 1.00E+03 |  | Number of iterations of the whole dataset. Following values were used: 10, 250, 500, 750, 1000. |
|  | MaxW² | 10 | 3.40E+38 |  | Constraint for squared sum of incoming weights per unit. The following values were used: 10, 100, 1000, 3.4028235e+38 (default). |
|  | Balanced classes |  |  |  | Balanced classes set to TRUE in order to balance the class distribution by either oversample or undersample the minority class. |
| RF | Number of trees | 1000 | 20000 | "+" 100 | Number of trees used to build the random forest. |
|  | Tree depth | 2 | 30 | "+" 1 | Maximum tree depth of a single tree. |
|  | Random variable selection | 1 | #predictors | "+" 1 | Number of variables randomly sampled as candidates at each split. |
|  | Sample rate | 0.1 | 1 | "+" 0.02 | Row sample rate for building a tree. |
|  | Balanced classes |  |  |  | Balanced classes set to TRUE in order to balance the class distribution by either oversample or undersample the minority class. |
| GBM | Number of trees | 1000 | 20000 | "+" 100 | Number of trees used for the additive sequence. |
|  | Tree depth | 2 | #predictors | "+" 1 | Maximum tree depth of a single tree. |
|  | Learning rate | 0.001 | 0.1 | "+" 0.001 | The learning rate defines the weight of each tree in the additive sequence. |
|  | Learning rate annealing | 0.9 | 0.999 | "+" 0.001 | Scaling factor of the learning rate after each tree. |
|  | Sample rate | 0.1 | 1 | "+" 0.02 | Row sample rate for building a tree. |
|  | Balanced classes |  |  |  | Balanced classes set to TRUE in order to balance the class distribution by either oversample or undersample the minority class. |
| MARS | nprune | 2 | 100 | "+" 1 | Maximum number of terms (including intercept) in the pruned model. |
| ELNET | Alpha | 0 | 1 | "+" 0.01 | Parameter for the distribution of regularization between the L1 (Lasso) and L2 (Ridge) penalties. |
|  | Lambda |  |  |  | R package "h2o" compiles an own sequence of potential lambda values, i.e. the regularization strength (max. n = 100). |

| **Variable** | **AUC** |
| --- | --- |
| Mean autumn water temperature | 0.90 |
| Water temperature of the coldest month | 0.90 |
| Maximum water temperature | 0.89 |
| Mean winter water temperature | 0.89 |
| Annual mean water temperature | 0.89 |
| Mean spring water temperature | 0.89 |
| Minimum water temperature | 0.88 |
| Water temperature of the warmest month | 0.82 |
| Built-up area | 0.81 |
| Annual water temperature range | 0.79 |
| Water temperature seasonality | 0.76 |
| Mean summer water temperature | 0.75 |
| Forest | 0.73 |
| Mean diurnal range | 0.67 |
| Minimum discharge | 0.65 |
| Discharge seasonality | 0.65 |
| Mean winter discharge | 0.65 |
| Flow of the driest month | 0.65 |
| Cropland | 0.64 |
| Grassland | 0.64 |
| Mean spring discharge | 0.64 |
| Mean autumn discharge | 0.61 |
| Isothermality | 0.60 |
| Annual mean discharge | 0.59 |
| Altitude | 0.59 |
| Shrubland | 0.57 |
| Maximum discharge | 0.51 |
| Flow of the wettest month | 0.50 |
| Mean summer discharge | 0.49 |

# Table S6 Univariate analysis of *Salmo trutta* distributions using generalized additive models.

# Table S7 Univariate analysis of *Salmo salar* distributions using generalized additive models.

| **Variable** | **AUC** |
| --- | --- |
| Mean winter water temperature | 0.89 |
| Maximum water temperature | 0.87 |
| Water temperature of the coldest month | 0.87 |
| Mean autumn water temperature | 0.87 |
| Water temperature of the warmest month | 0.87 |
| Mean spring water temperature | 0.86 |
| Annual mean water temperature | 0.86 |
| Minimum water temperature | 0.85 |
| Mean summer water temperature | 0.81 |
| Water temperature seasonality | 0.79 |
| Built-up area | 0.79 |
| Annual water temperature range | 0.78 |
| Forest | 0.74 |
| Minimum discharge | 0.73 |
| Flow of the driest month | 0.72 |
| Mean winter discharge | 0.70 |
| Isothermality | 0.66 |
| Altitude | 0.64 |
| Mean diurnal range | 0.62 |
| Shrubland | 0.60 |
| Cropland | 0.60 |
| Grassland | 0.57 |
| Discharge seasonality | 0.51 |
| Maximum discharge | 0.49 |
| Flow of the wettest month | 0.47 |
| Mean summer discharge | 0.45 |
| Annual mean discharge | 0.41 |
| Mean autumn discharge | 0.40 |
| Mean spring discharge | 0.36 |

# Table S8 Final tuning parameters for *Salmo trutta* distribution models (Artificial Neural Networks (ANN, manual and ADADELTA), Random Forest (RF), Gradient Boosting Machines (GBM), Multivariate Adaptive Regression Splines (MARS), Elastic Net (ELNET)) and the corresponding performance measures. 5-fold cross validation (cv) using 80% of the data was used for the parameter tuning. After final parameter selection, the final model prediction performance was tested for the remaining 20% of the data (test).

| **Method** | **Parameter** | **Final selection** | **AUC (cv)** | **AUC (test)** |
| --- | --- | --- | --- | --- |
| ANN  (manual) | Activation function | Rectifier | 0.9787 | 0.9795 |
|  | Number of nodes | 475 |  |  |
|  | Learning rate | 0.037 |  |  |
|  | Learning rate annealing | 1.00E-05 |  |  |
|  | Learning rate decay factor | 0.85 |  |  |
|  | Initial momentum | 0.4 |  |  |
|  | Final momentum | 0.97 |  |  |
|  | Dropout ratio | 0.2 |  |  |
|  | L1-regularization | 9.30E-05 |  |  |
|  | L2-regularization | 4.40E-05 |  |  |
|  | Epochs | 1003 |  |  |
|  | MaxW² | 3.40E+38 |  |  |
|  | Balanced classes | TRUE |  |  |
| ANN  (ADADELTA) | Activation function | Maxout | 0.9753 | 0.9754 |
|  | Number of nodes | 165 |  |  |
|  | Rho | 0.997 |  |  |
|  | Epsilon | 1.00E-09 |  |  |
|  | Dropout ratio | 0.05 |  |  |
|  | L1-regularization | 3.70E-05 |  |  |
|  | L2-regularization | 6.00E-05 |  |  |
|  | Epochs | 754 |  |  |
|  | MaxW² | 100 |  |  |
|  | Balanced classes | TRUE |  |  |
| RF | Number of trees | 12,200 | 0.9816 | 0.9795 |
|  | Tree depth | 25 |  |  |
|  | Random variable selection | 3 |  |  |
|  | Sample rate | 0.94 |  |  |
|  | Balanced classes | TRUE |  |  |
| GBM | Number of trees | 4,900 | 0.9787 | 0.9859 |
|  | Tree depth | 8 |  |  |
|  | Learning rate | 0.027 |  |  |
|  | Learning rate annealing | 0.997 |  |  |
|  | Sample rate | 0.52 |  |  |
|  | Balanced classes | TRUE |  |  |
| MARS | nprune | 58 | 0.9518 | 0.9388 |
| ELNET | Alpha | 0.01 | 0.8235 | 0.8002 |
|  | Lambda | 0.00145 |  |  |

# Table S9 Final tuning parameters for *Salmo salar* distribution models (Artificial Neural Networks (ANN, manual and ADADELTA), Random Forest (RF), Gradient Boosting Machines (GBM), Multivariate Adaptive Regression Splines (MARS), Elastic Net (ELNET)) and the corresponding performance measures. 5-fold cross validation (cv) using 80% of the data was used for the parameter tuning. After final parameter selection, the final model prediction performance was tested for the remaining 20% of the data (test).

| **Method** | **Parameter** | **Final selection** | **AUC (cv)** | **AUC (test)** |
| --- | --- | --- | --- | --- |
| ANN  (manual) | Activation function | Rectifier | 0.9728 | 0.9778 |
|  | Number of nodes | 410 |  |  |
|  | Learning rate | 0.002 |  |  |
|  | Learning rate annealing | 1.00E-06 |  |  |
|  | Learning rate decay factor | 0.85 |  |  |
|  | Initial momentum | 0.2 |  |  |
|  | Final momentum | 0.94 |  |  |
|  | Dropout ratio | 0.05 |  |  |
|  | L1-regularization | 8.00E-06 |  |  |
|  | L2-regularization | 0 |  |  |
|  | Epochs | 252 |  |  |
|  | MaxW² | 10 |  |  |
|  | Balanced classes | TRUE |  |  |
| ANN  (ADADELTA) | Activation function | Rectifier | 0.9649 | 0.9834 |
|  | Number of nodes | 75 |  |  |
|  | Rho | 0.998 |  |  |
|  | Epsilon | 1.00E-08 |  |  |
|  | Dropout ratio | 0.1 |  |  |
|  | L1-regularization | 8.60E-05 |  |  |
|  | L2-regularization | 6.80E-05 |  |  |
|  | Epochs | 502 |  |  |
|  | MaxW² | 10 |  |  |
|  | Balanced classes | TRUE |  |  |
| RF | Number of trees | 4,700 | 0.9802 | 0.9779 |
|  | Tree depth | 27 |  |  |
|  | Random variable selection | 3 |  |  |
|  | Sample rate | 0.24 |  |  |
|  | Balanced classes | TRUE |  |  |
| GBM | Number of trees | 10,600 | 0.9773 | 0.9560 |
|  | Tree depth | 8 |  |  |
|  | Learning rate | 0.094 |  |  |
|  | Learning rate annealing | 0.971 |  |  |
|  | Sample rate | 0.1 |  |  |
|  | Balanced classes | TRUE |  |  |
| MARS | nprune | 96 | 0.9581 | 0.9734 |
| ELNET | Alpha | 0.38 | 0.8448 | 0.8668 |
|  | Lambda | 0.00018 |  |  |

# References

Beitinger, T. L., Bennett, W. A., & McCauley, R. W. (2000). Temperature tolerances of North American freshwater species exposed to dynamic changes in temperature. *Environmental Biology of Fishes*, 58, 237–275.

Elliott, J. M. (1981). Some aspects of thermal stress on freshwater teleosts. In Stress and Fish (A. D. Pickering, ed), pp. 209-245. Academic Press, London.

Garside, E.T. (1973). Ultimate upper lethal temperature of Atlantic salmon (Salmo salar L.). *Canadian Journal of Zoology*, 51, 898–900.

Grande, M., & Andersen, S. (1991). Critical thermal maxima for young salmonids. *Journal of Freshwater Ecology*, 6, 275–279.

Jonsson, B., & Jonsson, N. (2009). A review of the likely effects of climate change on anadromous Atlantic salmon Salmo salar and brown trout Salmo trutta, with particular reference to water temperature and flow. *Journal of Fish Biology*, 75, 2381– 2447.

Küttel, S., Peter, A., & Wüest, A. (2002). Temperaturpräferenzen und-limiten von Fischarten

Schweizerischer Fliessgewässer, Rhône Revitalisierung, Publikation Nummer 1, März, 41 p (in German).

Laize, C. L. R., Acreman, M. C., Schneider, C., Dunbar, M. J., Houghton-Carr, H., Flörke, M., & Hannah, D. M. (2014). Projected flow alteration and ecological risk for pan-European rivers. *River Research and Applications*, 30, 299–314.

Lee, R. M., & Rinne, J. N. (1980). Critical thermal maximum of five trout species in the southwestern United States. *Transactions of the American Fisheries Society*, 109, 632–635.

Markovic, D., Freyhof, J., & Wolter, C. (2012). Where Are All the Fish: Potential of Biogeographical Maps to Project Current and Future Distribution Patterns of Freshwater Species. *PLoS ONE*, 7(7): e40530.

Markovic, D., Carrizo, S., Freyhof, J., Cid, N., Szabolcs, L., Scholz, M., Kasperdius, H., & Darwall, W. (2014). Europe’s freshwater biodiversity under climate change: distribution shifts and conservation needs. *Diversity and Distributions*, 20, 1097–1107.

Markovic, D., Carrizo, S. F., Kärcher, O., Walz, A., & David, J. N. W. (2017). Vulnerability of European freshwater catchments to climate change. *Global Change Biology*, 23, 3567–3580.

McNyset, K. M. (2005). Use of ecological niche modelling to predict distributions of freshwater fish species in Kansas. *Ecology of Freshwater Fish*, 14, 243–255.

Ojanguren, A. F., & Braña, F. (2003). Thermal dependence of embryonic growth and development in brown trout. *Journal of Fish Biology*, 62, 580–590.

Otto, S. A., & Zahn, S. (2008). Temperatur‐ und Sauerstoff‐Toleranz ausgewählter Wanderfischarten der Elbe. Potsdam: Institut für Binnenfischerei e.V (in German).

Todd, C. D., Hughes, S. L., Marshall, T., MacLean, J. C., Lonergan, M. E., & Biow, E. M. (2008). Detrimental effects of recent ocean surface warming on growth condition of Atlantic salmon. *Global Change Biology*, 14, 1–13.

Trautwein, C., Schinegger, R., Schmutz, S. (2012). Cumulative effects of land use on fish metrics in different types of running waters in Austria. *Aquatic Sciences*, 74, 329– 341.

Wallace, J. (1993). Environmental considerations. pp. 127–143. In: K. Heen, R. Monahan and F. Utter (eds). Salmon aquaculture. Fishing News Books. Oxford.

Wenger, S.J., Isaak, D.J., Dunham, J.B., Fausch, K.D., Luce, C.H., Neville, H.M., Rieman, B.E., Young, M.K., Nagel, D.E., Horan, D.L. & Chandler, G.W. (2011a). Role of climate and invasive species in structuring trout distributions in the Interior Columbia Basin. *Canadian Journal of Fisheries and Aquatic Sciences*, 68, 988–1008.

Wenger, S.J., Isaak, D.J., Luce, C.H., Neville, H.M., Fausch, K.D., Dunham, J.B., Dauwalter, D.C., Young, M.K., Elsner, M.M., Rieman, B.E., Hamlet, A.F. & Williams, J.E. (2011b). Flow regime, temperature, and biotic interactions drive differential declines of trout species under climate change. *Proceedings of the National Academy of Sciences of the United States of America*, 108, 14175–14180.

Yoder, C. O. (2012). Development of a Database for Upper Thermal Tolerances for New England Freshwater Fish Species. MBI Technical Report MBI/2012‐4‐6.
